# Low cost, low tech SNP genotyping tools for resource-limited areas: Plague in Madagascar as a model

**DOI:** 10.1371/journal.pntd.0006077

**Published:** 2017-12-11

**Authors:** Cedar L. Mitchell, Voahangy Andrianaivoarimanana, Rebecca E. Colman, Joseph Busch, Heidie Hornstra-O’Neill, Paul S. Keim, David M. Wagner, Minoarisoa Rajerison, Dawn N. Birdsell

**Affiliations:** 1 The Pathogen and Microbiome Institute, Northern Arizona University, Flagstaff, Arizona, United States of America; 2 Plague Unit, Institut Pasteur de Madagascar, Antananarivo, Madagascar; 3 Translational Genomics Research Institute, Flagstaff, Arizona, United States of America; University of New Hampshire, UNITED STATES

## Abstract

**Background:**

Genetic analysis of pathogenic organisms is a useful tool for linking human cases together and/or to potential environmental sources. The resulting data can also provide information on evolutionary patterns within a targeted species and phenotypic traits. However, the instruments often used to generate genotyping data, such as single nucleotide polymorphisms (SNPs), can be expensive and sometimes require advanced technologies to implement. This places many genotyping tools out of reach for laboratories that do not specialize in genetic studies and/or lack the requisite financial and technological resources. To address this issue, we developed a low cost and low tech genotyping system, termed agarose-MAMA, which combines traditional PCR and agarose gel electrophoresis to target phylogenetically informative SNPs.

**Methodology/Principal findings:**

To demonstrate the utility of this approach for generating genotype data in a resource-constrained area (Madagascar), we designed an agarose-MAMA system targeting previously characterized SNPs within *Yersinia pestis*, the causative agent of plague. We then used this system to genetically type pathogenic strains of *Y*. *pestis* in a Malagasy laboratory not specialized in genetic studies, the Institut Pasteur de Madagascar (IPM). We conducted rigorous assay performance validations to assess potential variation introduced by differing research facilities, reagents, and personnel and found no difference in SNP genotyping results. These agarose-MAMA PCR assays are currently employed as an investigative tool at IPM, providing Malagasy researchers a means to improve the value of their plague epidemiological investigations by linking outbreaks to potential sources through genetic characterization of isolates and to improve understanding of disease ecology that may contribute to a long-term control effort.

**Conclusions:**

The success of our study demonstrates that the SNP-based genotyping capacity of laboratories in developing countries can be expanded with manageable financial cost for resource constraint laboratories. This is a practical formula that reduces resource-driven limitations to genetic research and promises to advance global collective knowledge of infectious diseases emanating from resource limited regions of the world.

## Author summary

Although genetic characterization of pathogenic organisms is a powerful tool for investigating outbreak origins and transmission, associated high upfront costs and demanding technological maintenance exclude this tool for many under-resourced laboratories. Paradoxically, resource constrained regions commonly suffer from high rates of infectious diseases and could benefit most from genetic tracking tools. One such country is Madagascar, which lacks resources to acquire high tech genetic typing equipment, yet suffers from seasonal human plague outbreaks. A serious disease, plague is caused by the clonal bacterium, *Yersinia pestis*, and is capable of causing human outbreaks. Using plague as a model organism, we developed a genetic typing method that requires only basic, widely used molecular machinery. Our tools target unique single mutations in the *Y*. *pestis* genome to assign isolates to distinct phylogenetic groups with known geographical distributions. Transfer of this technology to Madagascar permits genetic characterization of strains from current outbreaks. This eliminates the need for external genetic analysis and expands the research capacity of this resource-constrained laboratory by allowing rapid, in-house strain typing. Ultimately, our goal is to help improve the ability of local institutes to genetically characterize circulating strains, link outbreaks to originating sources, and improve our understanding of the ecology of tropical diseases in resource-limited regions of the world.

## Introduction

Single nucleotide polymorphisms (SNPs) are highly valuable genetic markers that have advanced our knowledge of diverse biological fields such as human health [1,2], infectious disease epidemiology [3–5], agriculture [6], and ecology [7], among others. In the study of infectious diseases SNPs can be informative of bacterial phenotype, such as antibiotic susceptibility [8,9], and also can be used to classify unknown strains. For non-recombining bacterial pathogens, most of their SNPs become fixed in the genome and are faithfully replicated throughout future generations [3,10]. These stable signatures can be used to classify unknown strains into known phylogenetic groups according to SNP profiles [3,11,12]. Within the context of epidemiological investigations, these SNP profiles can link isolates from active outbreak sites to possible sources and help track disease transmission patterns [5,13,14].

Genotyping assays that use real-time PCR to identify single SNPs remain in demand despite the wide-scale availability of whole genome sequence (WGS) data and continued reductions in WGS costs. For many research facilities that are interested in small-scale studies or face resource limitations, a WGS-based approach to SNP typing is not a feasible nor a desirable option. A variety of other technological platforms have been employed for SNP typing and have been extensively described in several publications [15–18]. Popular platforms for SNP typing use real-time PCR instruments that employ Dual Probe TaqMan assays or melt-MAMA SNP assays [19–21]. But real-time platforms are not commonly available in resource constrained laboratories, due to their high upfront costs and the need for ongoing highly technical instrument maintenance. However, a more simplified method for SNP genotyping that employs conventional PCR coupled with standard agarose gel electrophoresis (agarose-MAMA) is a viable alternative in these settings. The advantage of this alternative method is that it utilizes relatively inexpensive instruments that are almost universally available even in developing nations where it is used for a variety of molecular applications. Much of this is due to the simplicity of the agarose gel electrophoresis platform, in contrast to the complex instrumentation of the real-time platform [20,22].

To illustrate the effectiveness of agarose-MAMA as a SNP genotyping tool in resource constrained laboratories, we developed *Y*. *pestis* assays for use at the Institut Pasteur de Madagascar (IPM). *Y*. *pestis* is the bacterium infamously known as the causal agent of the disease plague. *Y*. *pestis* is ecologically established on nearly every inhabited continent [12,23] and remains a particularly significant threat to human health in developing nations in Africa and especially the island country of Madagascar [24,25]. Primarily a zoonotic agent, *Y*. *pestis* has a complex ecological cycle involving rodent-host populations and flea vectors. In unfortunate circumstances, humans are incidental hosts [26]. Without prompt antibiotic treatment, human death rates vary from 30–60% to 100% depending on the route of exposure, with pneumonic plague being the most deadly [24,25]. Within the last two decades, Madagascar has reported some of the highest incidences of human plague infections throughout the world [24]. Given that *Y*. *pestis* is a re-emerging public health threat in Madagascar [27–29] and other developing African nations, performing SNP typing studies through the use of agarose-MAMA tools could lead to greatly improved epidemiological investigations and to more effective disease management.

Here, we describe how we resolved technological limitations that prevented *Y*. *pestis* SNP genotyping studies at IPM by: 1) re-designing a SNP-based *Y*. *pestis* genotyping system (from melt-MAMA real-time platform to agarose-MAMA) to be compatible with existing resources in Madagascar, and 2) validating these genetic tools at IPM for adoption. Collectively, these achievements have removed a research barrier at IPM once imposed by technological disparities and serve as a promising model for building similar research capacities in other developing nations affected by plague and/or other pathogens common to low-resource settings. In addition, these tools generate data that can be compared to other sequence-based methods and can feed into existing global databases. This ability can strengthen scientific exchange between affected countries and the international scientific community, thereby advancing the global collective knowledge of dangerous infectious diseases.

## Results and discussion

### Removal of technological barriers: Agarose MAMA–a highly accurate genetic typing tool

We present a SNP genotyping tool that uses standard genetic equipment and reagents that are accessible to nearly any laboratory. The successful development of agarose-MAMA tools removes the dependence on real-time instruments for conducting SNP-based epidemiological studies. Due to emerging infectious diseases being most globally prevalent in resource challenged countries [30,31], building research capacities in these regions has been a goal for the World Health Organization and other agencies charged with global biosafety, response, and biosecurity efforts [32,33]. It is now possible for researchers in resource constrained locations to conduct SNP studies to obtain bacterial phenotype information such as antibiotic resistance or phylogenetic information to more robustly understand the dynamics of disease transmission and potentially identify sources of human infections.

**Fig 1:**
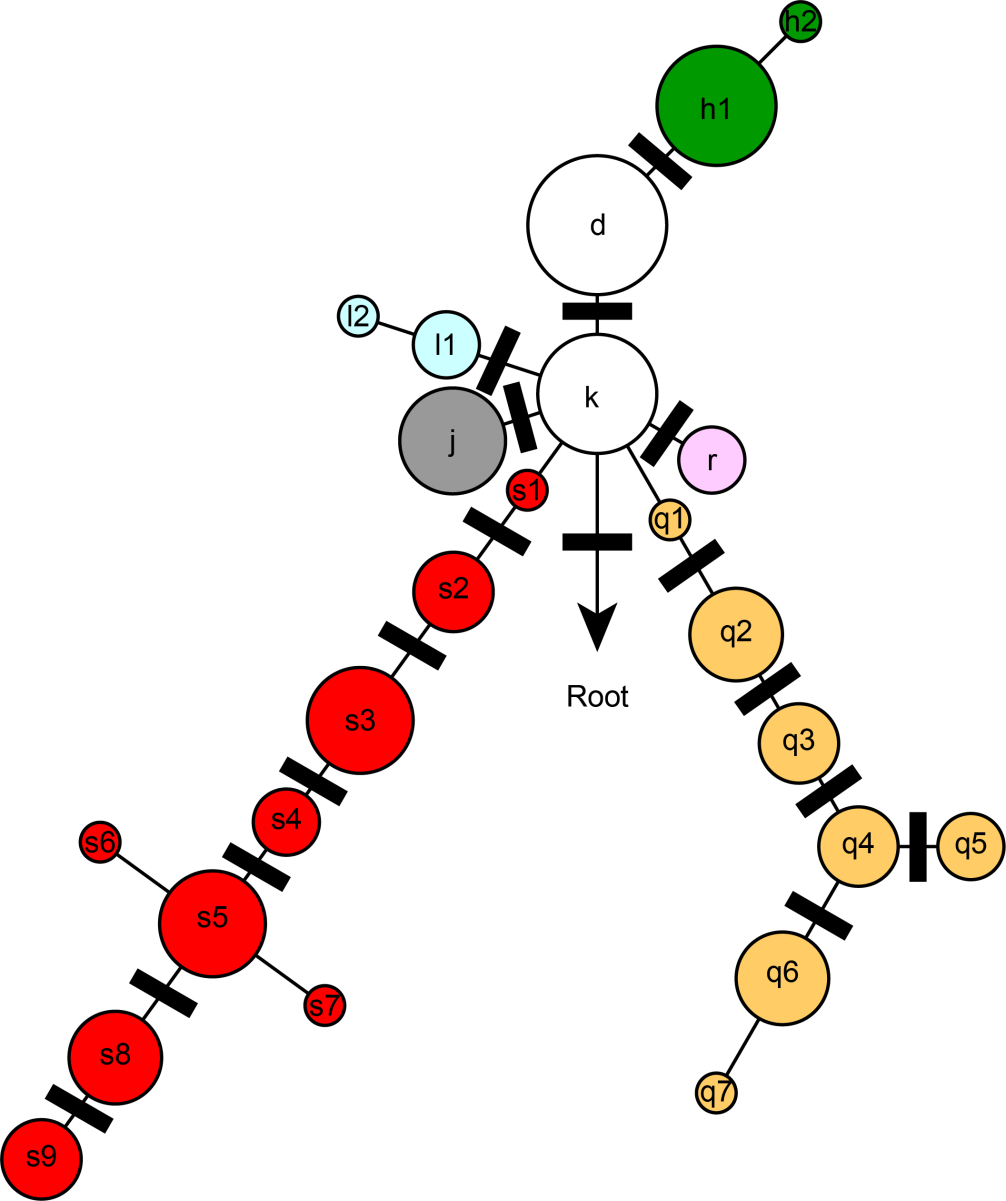
Simplified SNP phylogeny of *Y*. *pestis* depicting subgroups identified in Madagascar. Colored circles indicate phylogenetic groups as previously described [35][11,34]. Group names are assigned as letters and sometimes followed by a number (e.g. s4). Black bars indicate the phylogenetic positions of the 18 SNPs targeted for MAMA PCR design in this study.

**Fig 2:**
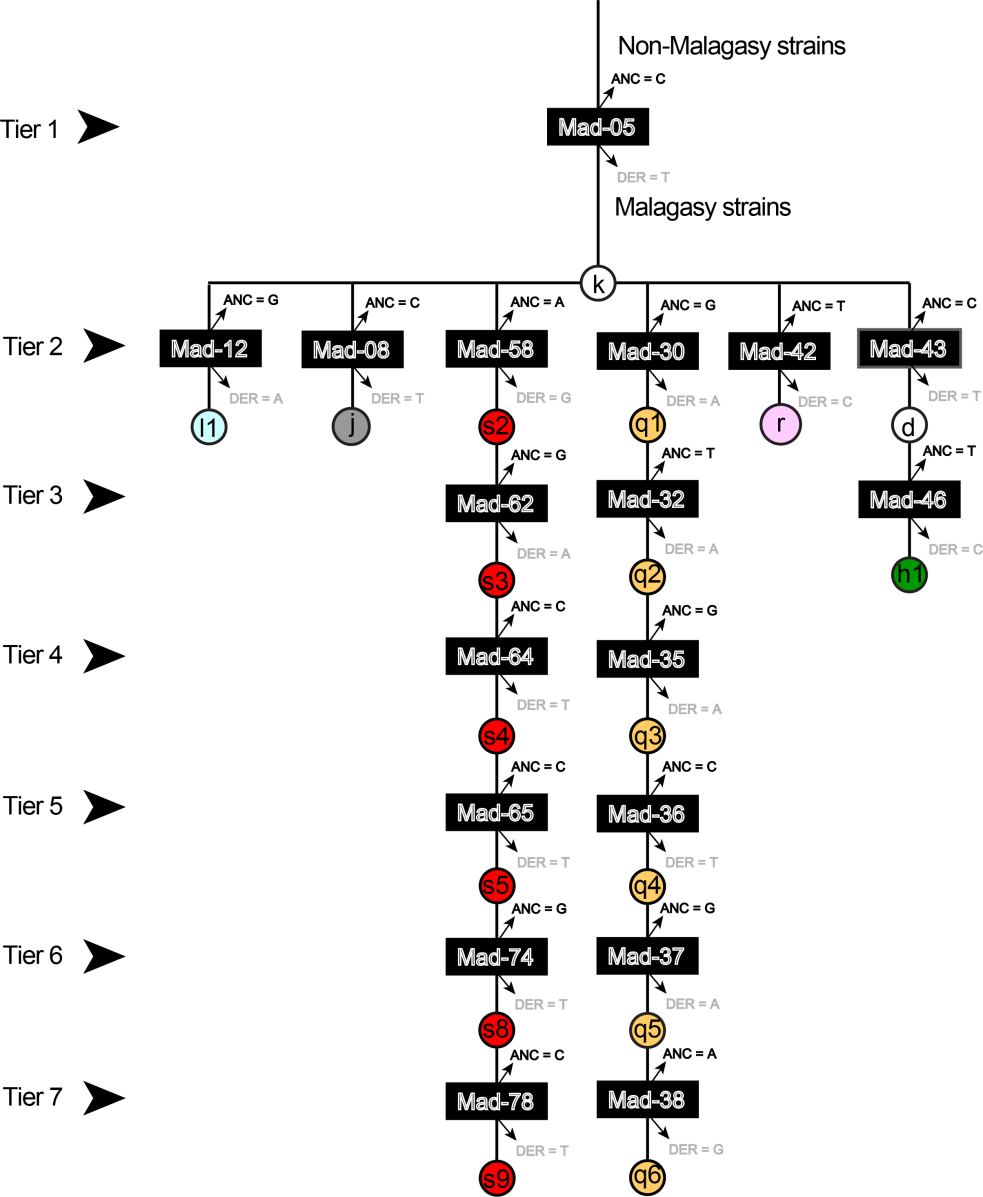
Flow chart providing the hierarchical organization of 18 SNP-genotyping assays. The hierarchy provides the sequential order to allow for stepwise identification of the genotype of an unknown isolate. Genetic subgroups are represented by colored circles labeled with each genetic subgroup. Individual assays are represented by the black bar labeled with each assay ID. The derived and ancestral allele states for each assay are indicated immediately above and below the black bar.

The phylogeny of *Y*. *pestis* in Madagascar has recently expanded [34] to include more phylogenetic groups (Fig 1) and the 18 assays developed for this study define a subset of these phylogenetic groups or lineages as illustrated on a simplified phylogeny (Fig 1). The genotyping results generated from each agarose-MAMA was identical to independent SNP genotyping technologies [34] when tested across the same diverse panel of 16 *Y*. *pestis* DNA strains. Assay specificity remained intact when these agarose-MAMAs were tested on diverse types of negative controls (high levels of human, *Leptospira* spp., and *Bacillus anthracis* DNA, or a no template water control). When the assays were employed in a stepwise, hierarchical order (Fig 2), each isolate could be assigned to a single lineage or one subgroup as depicted in the simplified Malagasy *Y*. *pestis* phylogeny (Fig 1). The equivalent performance of our agarose-MAMA tools to melt-MAMAs [11] and other independent SNP technologies [34] demonstrate that MAMA tools preserve their genotyping accuracy independent of the technology platform used.

### Optimization of assays to obtain accurate genotypes

For the 18 selected SNPs, we were able to successfully design agarose-MAMAs to achieve 100% genotyping accuracy through a few key optimization steps. Only one of our assays failed initial optimization efforts; however, when we redesigned the assay to target the reverse complement of the reference template DNA (strain CO92), the assay was rescued to full functionality. Essential validation steps included the identification of the optimal ratios for the concentration of the two forward primers (ancestral:derived) as described [20], ideal annealing temperature for each assay, appropriate number of PCR cycles, and an occasional need for MgCl_2_ concentration alteration in the reaction mix. These optimization strategies worked well on 8 other bacterial pathogens [20] and should be applicable to many other pathogenic organisms as well.

**Fig 3:**
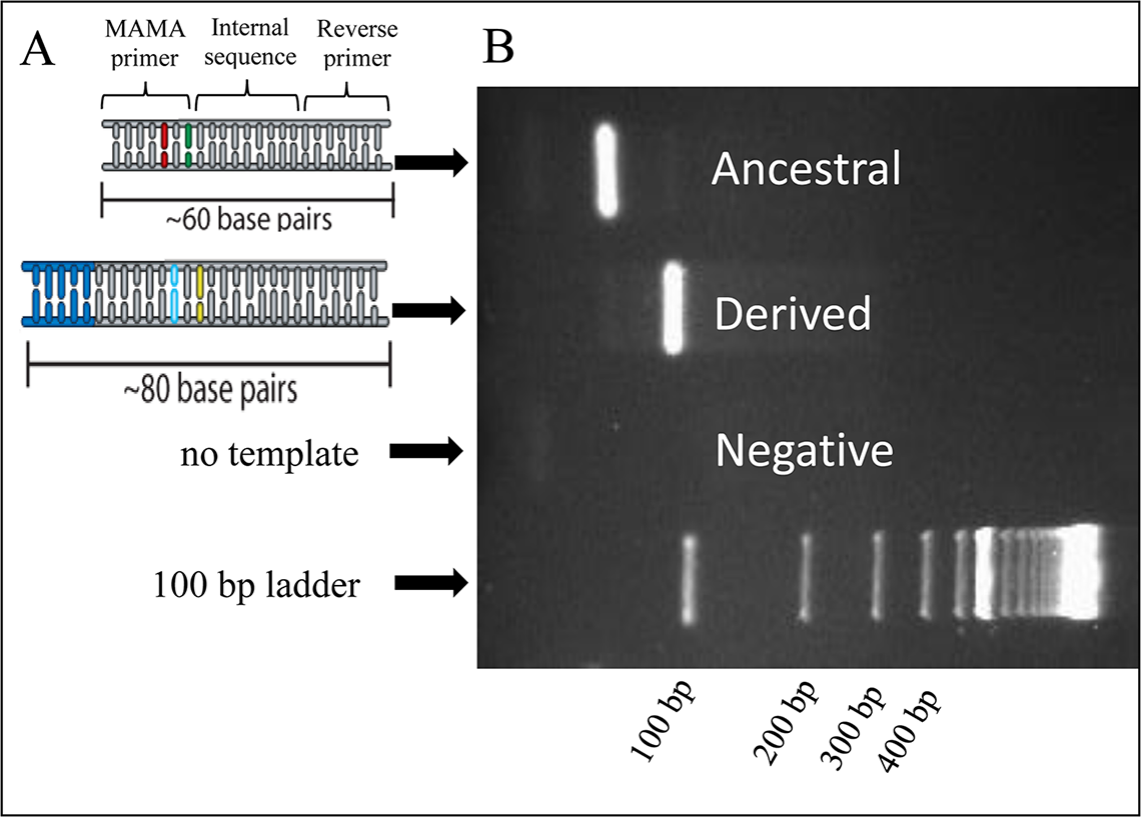
Size differences in allele-specific PCR products. A) Schematic of PCR amplicons originating from ancestral and derived genomic templates. For both PCR amplicons, gray represents sequence originating from the primers (forward and reverse) and synthesized internal sequence from PCR extension. The primer sequence and synthesized internal sequence within the amplicon are indicated by the bracket. For the derived amplicon, the blue represents the incorporated GC-clamp which originates from the 5’end of the derived MAMA forward primer. The SNP region for each amplicon is represented in green (ancestral) and yellow (derived) and the deliberate antepenultimate mutation is represented as red (ancestral) and light blue (derived). B) Allele-specific PCR products migrate at different rates on a 2% agarose gel due to their size difference as conferred by the GC-clamp.

The GC-clamp (21–28 oligo base), added to derived MAMA forward primers but excluded from ancestral MAMA forward primers, was sufficient to provide visible size differences between the two allelic states when visualized on an agarose gel (Fig 3). A repeated pattern of 5’-cgggttcgggttcgggttcgggttcggg-3’did not appear to induce non-specific binding nor interact with template DNA in an inhibitory manner. However, we observed that the GC-clamp on the derived MAMA primer did frequently confer a competitive advantage over the ancestral MAMA, as previously described for MAMA tools [20]. This competitive advantage resulted in cross-hybridization of primers for a subset of assays. This phenomenon may be based on the ability for GC rich DNA regions to anneal at lower temperatures compared to GC poor regions. The GC-clamp of the derived MAMA primer likely anneals to its target amplicon at lower temperatures compared to the no-clamp ancestral primer. As a consequence, the GC-clamp primer anneals to the template at an earlier time point per PCR cycle than the no-clamp primer, resulting in a competitive kinetic advantage. To correct for this competitive advantage, we followed published guidelines [20] by increasing the ancestral primer concentration relative to the derived primer concentration of the affected assays. In these skewed reaction mixes, the ancestral primer had >1x-4x concentration compared to its paired derived primer. We have detected best results with 4:1 and 2:1 (ancestral:derived) primer ratios, depending on the assay.

We obtained maximal assay specificity by a combination of customizing the annealing temperature per assay and/or adjusting the MgCl_2_ concentrations. Most of our assays accurately genotyped SNP alleles at annealing temperatures around 60°C; however, some assays had non-specific product at this condition. For these assays, non-specific PCR products were present in our negative controls and/or in our sample PCR product, visualized as extra banding on a gel. A reduction of MgCl_2_ concentration by 0.5 mM was sufficient to greatly increase the specificity of most assays, as evidenced by the elimination of non-specific amplification. But this reduction in MgCl_2_ decreased the PCR robustness in a subset of assays. To address this loss, the annealing temperatures were reduced in affected assays while still preserving SNP specificity. For the few assays that did not respond to lowered MgCl_2_ strategy, the raising of annealing temperatures while still maintaining the normal 2.0 mM concentration of MgCl_2_ yielded improvements in reducing non-specific products.

### Assay function on clinical samples

**Fig 4:**
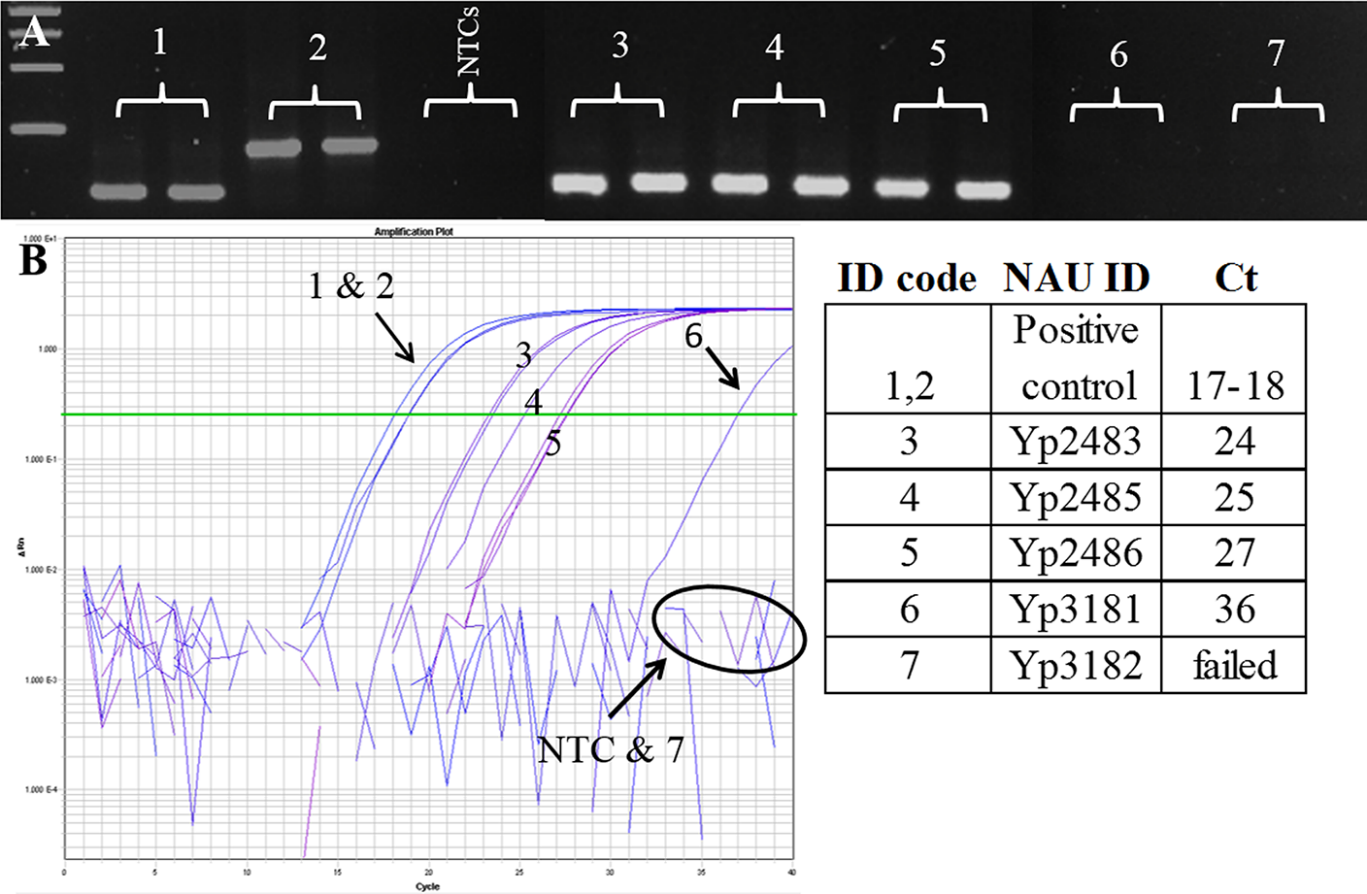
Agarose-MAMA is capable of genotyping *Y*. *pestis* directly from complex clinical samples if pathogen targets are at sufficient levels. (A) Agarose-MAMA (Mad-43) gel showing the PCR products for two template controls at the expected size for each respective ancestral and derived allele state (#1 and #2, respectively). Three complex clinical samples (#3-#5) also yielded PCR products with the size expected of an ancestral genotype. Two other complex clinical samples (#6 & #7) showed no PCR products but displayed a banding pattern consistent with NTC negative controls. (B) To assess the relative quantity of the *Y*. *pestis* target in the five clinical samples (#3, #4, #5, #6, #7), we generated amplification plots of these clinical samples on a TaqMan 3a assay used to target *Y*. *pestis* chromosomal DNA. Three clinical samples (#3-#5) showed amplified at a mid-range cycle-time (Ct) value consistent with high copy numbers of template DNA [21]. These same samples showed a robust signal of a PCR product on the MAMA gel. The two samples (#6 & #7) that failed on the MAMA gel showed a high Ct value and failed amplification, respectively, when tested by real-time PCR. The pairing of TaqMan 3a assay with MAMA gel results on the same templates provided insight to the genotyping capability of MAMA tools on complex clinical samples with low-level target template.

The assays described here are capable of genotyping directly from complex clinical samples if pathogen DNA levels are sufficient (Fig 4). This capability is highly important as nearly 43.32% of 775 F1 RDT [36] positive human plague cases between 2011–2015 in Madagascar and many environmental samples (rodents and fleas) do not yield live isolate culture (IPM records). Using two agarose MAMA tools (Mad-05 and Mad-43) to demonstrate proof of principal, we were able to determine that the three complex clinical samples belonged to group I lineage and not group II. This genetic assignment for these samples matched the results of a recent publication [34]. These samples were not further tested on additional agarose-MAMAs. Out of five complex clinical samples positive for the high copy plasmid *pla* gene [37] only four were positive for *Y*. *pestis* chromosomal DNA, assessed by a TaqMan assay targeting the *3a* gene (Fig 4 and S3 Appendix). Of the four *3a*-positive samples, one isolate (Yp3182) gave a late amplification with the 3a assay resulting in a Ct value of 36 using real-time PCR. This indicates a very low concentration amount for this clinical extract, near a single copy of pathogen DNA [20,21]. The other three samples (Yp2483, Yp2486, Yp2485) (Fig 4) generated Ct values ranging from 24–27, which indicates higher concentrations of *Y*. *pestis* chromosomal DNA in these clinical samples. These more concentrated *Y*. *pestis* clinical samples were successfully genotyped by agarose-MAMA tools following an increase in the number of PCR cycles. The low-level sample (Yp3182) failed to amplify PCR product using agarose-MAMA tools. Together, these results indicate that agarose-MAMAs can genotype directly from complex clinical samples if the pathogen target (*Y*. *pestis* DNA) is of sufficient concentration. Published studies show that TaqMan assays are highly sensitive [20,21] and can readily detect minute amount of template not detectable using melt-MAMAs [20]. Our results suggest that the same is true for agarose-MAMAs (Fig 4).

### Transfer of technology–the minor effects of different research facilities and reagents

**Fig 5:**
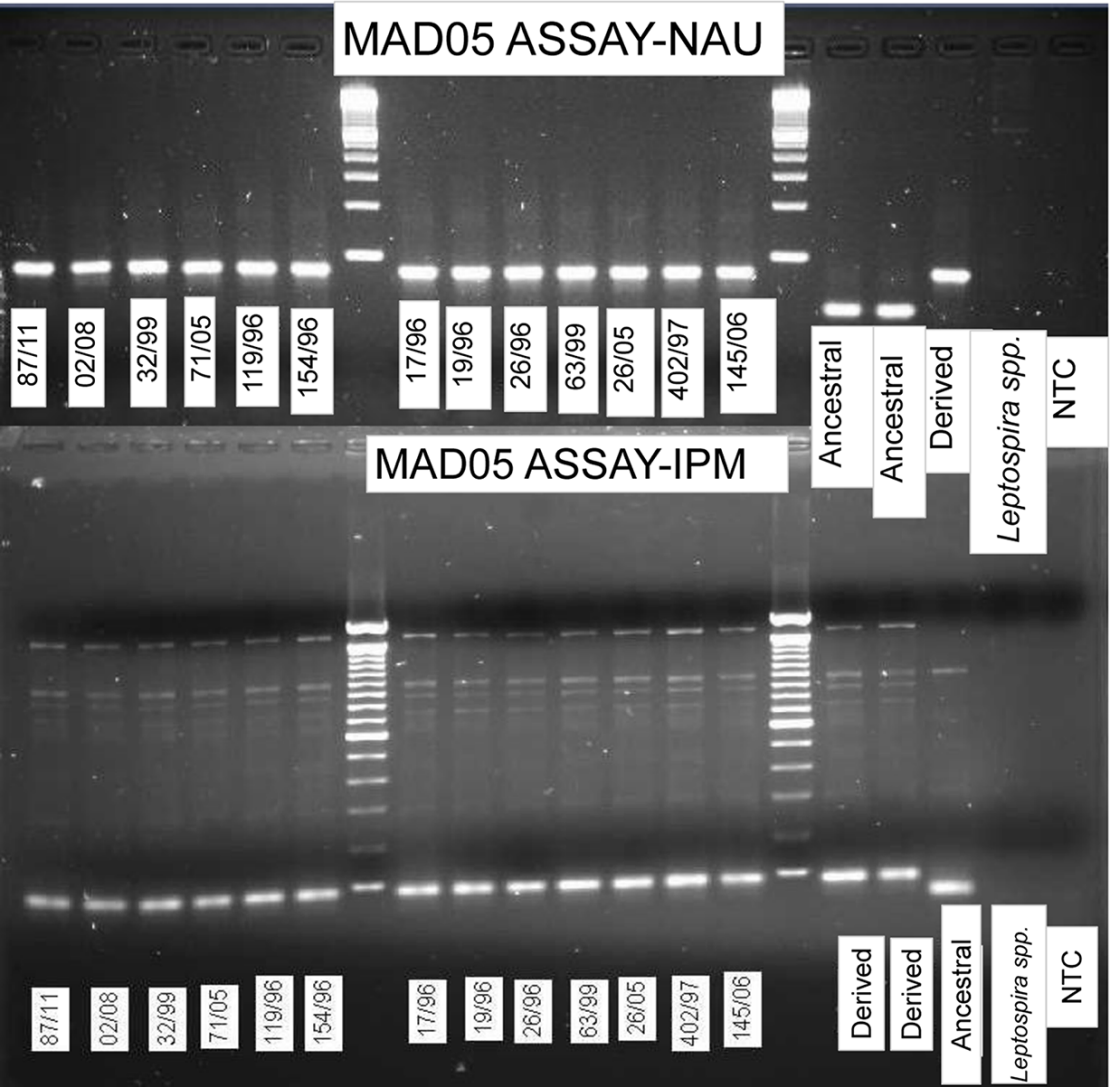
Mad-05 MAMA tool showed congruent genotyping results when generated across two research institutes using identical DNA samples. Side by side comparison of Agarose-MAMA gel images generated at NAU (top) and IPM (bottom) using different reagents and instruments. *Y*. *pestis* sample names follow the designation scheme practiced at IPM. Individual samples are designated an ID # according to consecutive order of collection per a given year. Strain 87/11 was the 87^th^ isolate collected in 2011.

Following the transfer of our agarose-MAMA tools to the IPM facility, a subset of our agarose-MAMAs was validated for genotyping accuracy. The differing instruments and reagents used at IPM introduced very little variability on assay performance when tested on the same DNA panel previously used at NAU (Fig 5). The optimized PCR conditions identified at NAU for the assays were suitable for most of the assays when conducted at IPM.

The most apparent difference in assay performance between the two laboratories was an increase in non-specific amplification at high fragment size (Fig 5) and, for some assays, also in our negative controls at IPM, which was not observed at NAU. These non-specific fragments did not affect the MAMA’s capability to accurately genotype isolates. Band profiles were not distinctly in line with either the ancestral or derived product but rather appeared either as a smear across both profiles or as a much fainter band falling between the ancestral and derived fragment sizes. We suspect that the basis of this performance difference is due to different *Taq* polymerases used between the two institutions. NAU employed an antibody-immobilized *Taq* polymerase (Invitrogen, Carlsbad, CA), which has been characterized as having no polymerase activity prior to a hot-start step in the thermal cycle protocol [38]. However, IPM utilizes regular *Taq* polymerase, which may begin product synthesis with primers and template in the master mix prior to PCR thermal cycling [38]. Although master mix preparation was done on ice to suppress premature *Taq* polymerase activity, additional bands above the target PCR product and faint bands in the NTC amplification suggest that pre-PCR *Taq* activity was not completely suppressed (Fig 5). We therefore reduced the concentration of MgCl_2_ in five of our assays (Mad-43, Mad-46, Mad-12, Mad-36 and Mad-58) to 1.5mM and observed the elimination of amplification in the negative controls in most of the affected assays. Reduction of MgCl_2_ also imparted a general improvement of assay specificity for positive control and test isolates. Surprisingly, the reduction of MgCl_2_ did not necessitate a corresponding increase in the number of PCR cycles for most assays, contrary to the results observed in our laboratory at NAU. Once again, this may be a result of differences in *Taq* polymerase activity between the two institutions although we have not found any published evidence of this occurring elsewhere. As a way to rule out possible contamination as a source of non-specific banding, all surfaces used for the preparation of PCR reaction mixtures were sterilized with UV light for 15 minutes and decontaminated with 70% ethanol. Following slight modifications, we found that results of the majority of our assays at IPM aligned very well with results produced at NAU (Fig 5 and S5 Appendix).

### Advancing global collective knowledge by building research capacity

Our success in developing agarose-MAMA tools and transferring them to IPM facility in Madagascar demonstrates that this SNP genotyping strategy can be achieved with existing technologies routinely used in developing nations (Fig 5). The transferred agarose-MAMA technology is now in regular use at IPM and was recently used to infer the source of a 2015 pneumonic outbreak [39]. There is ample evidence that this same assay design strategy would transfer well to many other pathogenic organisms [20]. This would allow institutions in developing countries to perform molecular studies in-house and on local infectious organisms. The current methods largely used for short-term control efforts are presence/absence assays (PCR based [40] or protein based [36]) that diagnose the causative agents of disease outbreaks but provide no genetic resolution. Having in-house capabilities to genetically discriminate goes beyond what presence/absence assays can provide, therefore, resource-constrained laboratories will be able to advance their epidemiological capabilities over the status quo. The SNP data they generate locally can be shared among research institutes and compared to existing global databases. These advanced capabilities would accelerate the understanding of plague ecology, persistence, and evolution; which in turn could beneficially inform strategies for disease control.

Building research capacity in low-resource settings is an important endeavor for global biosafety, response, and biosecurity preparedness [32,33]. Our study is a successful model of achieving this goal pragmatically. We successfully developed and transferred genetic tools to a developing nation. The design principles for this technology, which we detail above, can be applied to diverse pathogen species [20]. The use of MAMA technology will give the scientific community the means to gain insight into the genetic patterns and population structure of many neglected diseases. This is a practical formula that will advance global collective knowledge of infectious diseases emanating from more impoverished regions of the world.

## Materials and methods

### Ethics statement

The 16 archival strains and five clinical samples were not subject to IRB regulations because they did not meet the federal definition of human subjects research according to *45 CFR 46*.*102 (f)*. All samples underwent de-identification of patient information prior to Northern Arizona University (NAU) transfer. They were collected as part of the medical workup mandated by the Ministry of Health in Madagascar and not for the purpose of this study. For this reason the strains used in this study does not meet the federal definition of human subjects research according to *45 CFR 46*.*102 (f)* and therefore are not subject to review from NAU Institutional Review Board.

### DNA

DNA samples utilized in this study were obtained from 16 *Y*. *pestis* isolates and 5 clinical samples (bubo aspirates and sputum) collected from suspected human plague cases (Table 1). The 16 archival strains and 5 clinical samples (Table 1) originated from diverse geographic locations in Madagascar and were collected as described in a recently published study [34]. Our assays worked well on DNA concentrations that ranged from 1 ng to100 pg. Molecular confirmation of *Y*. *pestis* in five clinical samples was based on PCR detection of the *pla* gene located in a high copy number plasmid PCP1 in addition to positive F1 RDT [28,36,37,41,42].

**Table 1:** *Y*. *pestis* isolates from Madagascar used in this study.

ID	Original ID	DNA Type	pla status	3a status	Tested at IPM?	Group^*c*^	Vogler et al. 2011 SNP Node^*d*^	Vogler et al. 2017 SNP Node^*e*^	Originating Lab^*f*^	Province	District	Commune	Village	Source	Year	Previously published^*g*^
Yp2554	194/95	bacterial culture	+	+	yes	I	q05	q05	IPM	Antananarivo	Anjozorobe	Ambongamarina	Ambohimiaramanana	human	1995	Vogler et al. 2017
Yp2564	67/96	bacterial culture	+	+	yes	I	r	r	IPM	Antananarivo	Antsirabe II	Vinaninkarena	Fiakarandava	human	1996	Vogler et al. 2017
Yp2669	172/98	bacterial culture	+	+	yes	I	s9	s09	IPM	Mahajanga	Mahajanga I	Mahabibo	Abattoir	*Suncus murinus*	1998	Vogler et al. 2017
Yp2975	145/06	bacterial culture	+	+	yes	I	q2	q10	IPM	Toamasina	Moramanga	Andasibe	Ampangalantsary	human	2006	Vogler et al. 2017
Yp2610	402/97	bacterial culture	+	+	yes	I	q06	q06	IPM	Antananarivo	Antsirabe II	Ibity	Ampopoka	human	1997	Vogler et al. 2017
Yp2924	26/05	bacterial culture	+	+	yes	I	q06	q06	IPM	Antananarivo	Manjakandriana	Ambatomena	Antsahakely	human	2005	Vogler et al. 2017
Yp2559	17/96	bacterial culture	+	+	yes	II	d	v01	IPM	Antananarivo	Betafo	Mandritsara	Miadana	human	1996	Vogler et al. 2017
Yp2560	19/96	bacterial culture	+	+	yes	II	d	v01	IPM	Antananarivo	Betafo	Miadana Miaramasoand	Ambohijato	human	1996	Vogler et al. 2017
Yp2561	26/96	bacterial culture	+	+	yes	II	d	v02	IPM	Antananarivo	Betafo	Tritriva	Iavonarivo	human	1996	Vogler et al. 2017
Yp2568	119/96	bacterial culture	+	+	yes	II	h1	h11	IPM	Antananarivo	Antsirabe II	Mangarano	Maninarivo	human	1996	Vogler et al. 2017
Yp2572	154/96	bacterial culture	+	+	yes	II	h1	h11	IPM	Antananarivo	Antsirabe I	Antsenakely Andraikiba I	Andrangy	human	1996	Vogler et al. 2017
Yp2672	32/99	bacterial culture	+	+	yes	I	l1	l01	IPM	Antananarivo	Antsirabe II	Alatsinainy Ibity	Sahamalaza	human	1999	Vogler et al. 2017
Yp2675	63/99	bacterial culture	+	+	yes	I	s03	s03	IPM	Antananarivo	Betafo	Ambohimanambola	Vinanisoa	human	1999	Vogler et al. 2017
Yp2933	71/05	bacterial culture	+	+	yes	I	s05	s05	IPM	Antananarivo	Manjakandriana	Manjakandriana	Anosimanarivo	human	2005	Vogler et al. 2017
Yp3020	02/08	bacterial culture	+	+	yes	I	s05	s05	IPM	Antananarivo	Manjakandriana	Manjakandriana	Manakasikely	human	2008	Vogler et al. 2017
Yp3070	87/11	bacterial culture	+	+	yes	I	j	j10	IPM	Antananarivo	Betafo	Mandoto	Analavory	human	2011	Vogler et al. 2017
Yp2483^*a*^	644/M 004	complex clinical sample	+	+	no	I	k	k	IPM/BIM^b^	Fianarantsoa	Ambalavao	Sendrisoa	Manakony	human	2007	Riehm et al. 2015
Yp2486^*a*^	571/M 044	complex clinical sample	+	+	no	I	q3	q12	IPM/BIM^b^	Mahajanga	Tsaratanana	Keliloha	Ambatomitsangana	human	2007	Riehm et al. 2015
Yp2485^*a*^	645/M 017	complex clinical sample	+	+	no	I	s3	s13	IPM/BIM^b^	Antananarivo	Faratsiho	Ramainandro	Alatsinainy Bevohoka	human	2007	Riehm et al. 2015
Yp3181^*a*^	TLO432/13	complex clinical sample	+	-	no	unknown	unknown	unknown	IPM	Mahajanga	Mandritsara	Ampatakamaroreny	Beranimbo	human	2013	—
Yp3182^*a*^	TLO464/13	complex clinical sample	+	+	no	unknown	unknown	unknown	IPM	Mahajanga	Mandritsara	Antanambaon'amberina	Sahakondro	human	2013	—

^*a*^Strain ID in the Northern Arizona University DNA collection.

^*b*^Strain ID from the originating laboratory.

^*c*^Indicates Group I or II, as described (Vogler et al. 2011).

^*d*^Indicates SNP determined node (Vogler et al. 2011).

^*e*^Indicates SNP determined node (Vogler et al. 2017). For samples where a specific node could not be determined, the lineage letter is indicated without a specific node number.

^*f*^IPM: Institut Pasteur de Madagascar, BIM: Institut für Mikrobiologie der Bundeswehr.

^*g*^These samples were analyzed previously in the listed references.

### SNP groups

Eighteen previously published SNPs [11,34,35] specific, or canonical [3], for a subset of distinct phylogenetic groups within the *Y*. *pestis* Malagasy phylogeny (Fig 1) were selected as the targets of agarose MAMA genotyping assays following published guidelines [20]. These selected SNPs can be used in a hierarchical way (Fig 2; Table 2) to assign an unknown strain to one of the most common lineages or phylogenetic subgroups in Madagascar [11,34,35,43].

**Table 2:** The SNP allele state profiles of genetic subgroups targeted for agarose MAMA design where the Derived SNP state is shaded and Ancestral state is unshaded.

Major Group^*a*^	Subgroup^*a*^	Tier 1^*b*^	Tier 2 ^*b*^	Tier 2 ^*b*^	Tier 2 ^*b*^	Tier 3 ^*b*^	Tier 4 ^*b*^	Tier 5 ^*b*^	Tier 6 ^*b*^	Tier 7 ^*b*^	Tier 2 ^*b*^	Tier 3 ^*b*^	Tier 4 ^*b*^	Tier 5 ^*b*^	Tier 6 ^*b*^	Tier 7 ^*b*^	Tier 2 ^*b*^	Tier 2 ^*b*^	Tier 3 ^*b*^
		*Mad-05^*c*^ C→T	Mad-12 ^*c*^ G→A	Mad-08 ^*c*^ C→T	Mad-58 ^*c*^ A→G	Mad-62 ^*c*^ G→A	Mad-64 ^*c*^ T→C	Mad-65 ^*c*^ C→T	Mad-74 ^*c*^ G→T	Mad-78 ^*c*^ C→T	Mad-30 ^*c*^ G→A	Mad-32 ^*c*^ T→A	Mad-35 ^*c*^ G→A	Mad-36 ^*c*^ C→T	Mad-37 ^*c*^ G→A	Mad-38 ^*c*^ A→G	Mad-42 ^*c*^ T→C	Mad-43 ^*c*^ C→T	Mad-46 ^*c*^ T→C
I	k^*d*^	T	G	C	A	G	T	C	G	C	G	T	G	C	G	A	T	C	T
I	I1	T	A	C	A	G	T	C	G	C	G	T	G	C	G	A	T	C	T
I	j	T	G	T	A	G	T	C	G	C	G	T	G	C	G	A	T	C	T
I	s2	T	G	C	G	G	T	C	G	C	G	T	G	C	G	A	T	C	T
I	s3	T	G	C	G	A	T	C	G	C	G	T	G	C	G	A	T	C	T
I	s4	T	G	C	G	A	C	C	G	C	G	T	G	C	G	A	T	C	T
I	s5	T	G	C	G	A	C	T	G	C	G	T	G	C	G	A	T	C	T
I	s8	T	G	C	G	A	C	T	T	C	G	T	G	C	G	A	T	C	T
I	s9	T	G	C	G	A	C	T	T	T	G	T	G	C	G	A	T	C	T
I	q1	T	G	C	A	G	T	C	G	C	A	T	G	C	G	A	T	C	T
I	q2	T	G	C	A	G	T	C	G	C	A	A	G	C	G	A	T	C	T
I	q3	T	G	C	A	G	T	C	G	C	A	A	A	C	G	A	T	C	T
I	q4	T	G	C	A	G	T	C	G	C	A	A	A	T	G	A	T	C	T
I	q5	T	G	C	A	G	T	C	G	C	A	A	A	T	A	A	T	C	T
I	q6	T	G	C	A	G	T	C	G	C	A	A	A	T	A	G	T	C	T
I	r	T	G	C	A	G	T	C	G	C	G	T	G	C	G	A	C	C	T
II	d	T	G	C	A	G	T	C	G	C	G	T	G	C	G	A	T	T	C
II	h1	T	G	C	A	G	T	C	G	C	G	T	G	C	G	A	T	T	T

^*a*^Described in Vogler *et al* 2011 and Vogler *et al* 2017

^*b*^Corresponds to levels indicated in Fig 2 in this study.

^*c*^canSNP that define Malagasy subgroups.

^*d*^Collapsed node containing many subgroups.

### Assay design

The common reverse primer and two forward allele-specific MAMA primers for each assay were designed using NetPrimer analysis software (Premier Biosoft, Palo Alto, CA). One forward primer represents the original SNP allele, referred to as “ancestral” and the other represents the mutated SNP allele, referred to as “derived”. The MAMA primers for each assay were designed to compete for the same SNP locus on the template and the resulting amplicon product is generated by the allele-specific MAMA primer that most closely matches the template.

To differentiate between the amplicon products of the derived and ancestral genotypes, additional length was added on the derived amplicon product but not the ancestral product. This was achieved by adding 21–28 oligonucleotides rich in GC content at the 5’end of the derived MAMA forward primer (GC-clamp) (Fig 3). Since the primers are incorporated in the final amplicon product, the addition of the GC-clamp on the derived MAMA forward primer resulted in derived amplicon products that were 21–28 bp longer than their ancestral amplicon counterparts.

To maximize the visible size differences between the two allelic-specific amplicons when viewed on an agarose gel, the size of the PCR amplicon was restricted to ≤ 80 bases total length. Amplicons within ~80 bases show the greatest migration difference on a gel when small size differences exist. This is the case with our derived and ancestral allele-specific PCR products, which differ between 21–28 bases (Fig 3, Table 3, S2 Appendix). Additionally, through the use of the GC-clamp, our assays retained the capability of SNP genotype discrimination on a real-time PCR platform, which is based on differential melt-curve properties of each SNP-specific PCR product [20].

**Table 3:** Agarose-MAMA primers targeting 18 previously published SNP positions.

SNP^a^	CO-92 Position^a^	SNP State (A→D)^b^	Vogler *et al*. 2011 and 2013 SNP group	Vogler *et al*. 2017 SNP group	Primers^c^^,^^d^	^e^Conc. (μM)	NAU-Agarose Annealing temperature (°C) and Cycle Repeats	Facility site of validation
Mad-05	3525070	C→T	All	NA	A: GTGGCTGGCAGCGGTCtC D: cgggttcggggttcggggttcggggttcggggGTGGCTGGCAGCGGTCaT C: GAAGCTGAACAAAATGCGACTAATA	0.200 0.200 0.200	60°C 33 cycles	NAU and IPM
Mad-43	1348724	C→T	d/e/i/h1/h2	k-d	A: GGTTGCTGATGAACACGGtC D: cgggttcgggttcgggttcggggttcggggGGTTGCTGATGAACACGGcT C: GCAACCAAATCAGCAAAATAGAGA	0.200 0.200 0.200	60°C 33 cycles	NAU and IPM^g^
Mad-46	1791464	T→C	h1/h2	hu-h	A: ATACAGGATTATTTTAAATGGcA D: cgggttcggttcgggttcgggttcgggATACAGGATTATTTTAAATGGgG C: GCATAAGTATTGCAATTTAATTTC	0.200 0.200 0.200	55°C 33 cycles	NAU and IPM^g^
Mad-08	3210210	C→T	j	k-j01	A: CAGCTTCACGCGACGACtAC D: cgggttcgggttcggggttcggggttcggggAGCTTCACGCGACGACaAT C: GGCAGAGGAAGACCATCAACC	0.200 0.200 0.200	67.3°C 33 cycles	NAU and IPM
Mad-12	805205	G→A	l1/l2	k-l01	A: GGCTGTGGATGCGGGTaG D: cggggttcggggttcggggttcggggttcgggGGCTGTGGATGCGGGTtA C: GTTGTCCATCGGTAGCATCTTG	0.200 0.200 0.200	60°C 35 cycles	NAU and IPM^g^
Mad-42	3926933	T→C	r	k-r	A: ATAGTAACATACAGTAAAGTGACAATAAgT D: cgggttcggggttcgggttcgggGTAACATACAGTAAAGTGACAATAAcC C: CTAAATAGAGTGTACGCTTAACAAC	0.800 0.100 0.200	54°C 35 cycles	NAU and IPM^g^
Mad-30	2899068	G→A	q1-q7	k-qI	A: GCACGAAACGCCTCATGCGtCC D: cggggtttcggggtttcggggttttcggggACGAAACGCCTCATGCGaCT C: TGGTGTGTGGCGGCGGCGTG	0.800 0.200 0.200	60°C 31 cycles	NAU and IPM^g^ MgCl_2_ = 1.5mM
Mad-32	1632446	T→A	q2-q7	k-qI	A: AAACATAACCACCAGCCAAATAgT D: cgggttcgggttcgggttcgggttcgggACATAACCACCAGCCAAATcAA C: TGCGGGAGGGGCTTTACT	0.200 0.200 0.200	60°C 33 cycles	NAU and IPM^g^
Mad-35	3897676	G→A	q3-q7	qI-qII	A: GAGATGCTCAGCGAGCGAtG D: cgggttcggggttcggggttcggggttcggggGATGCTCAGCGAGCGgAA C: AAGGCAGATGCAACGGATAAC	0.200 0.200 0.200	^f^MgCl_2_ [1.5mM] 60°C 40 cycles	NAU and IPM^g^ MgCl_2_ = 1.5mM
Mad-36	3533308	C→T	q4-q7	qII-q04	A: ACCCGTGAGCAAAACCcC D: cggggttcggggtttcggggttcggggATTTAACCCGTGAGCAAAACaGT C: ATGGCCATAGCAAAGGTGACA	0.400 0.200 0.200	60°C 33 cycles	NAU and IPM^g^ MgCl_2_ = 1.5mM
Mad-37	606374	G→A	q5	q04-q05	A: ATCACCATCCCGAACGATtAG D: cggggtttcggggtttcggggtttcggggATCACCATCCCGAACGATAcA C: ACGCCCCAGAACTTTCAATAG	0.200 0.200 0.200	60°C 33 cycles	NAU and IPM^g^
Mad-38	1950363	A→G	q6/q7	q04-q06	A: GAATATCCAAGCGTTGCTGAgT D: cgggttcgggttcgggttcggggttcgggAATATCCAAGCGTTGCTGAcC C: TTGCTGGAAGGTGGAAATG	0.800 0.200 0.200	MgCl_2_ [2.5mM] 60°C 30 cycles	NAU and IPM^g^
Mad-58	2681067	A→G	s2-s9	k-s	A: AGATGTGGCCAAACACGgA D: cgggttcggggttcgggttcgggttcgggAAAGATGTGGCCAAACACGaG C: CGACGAAACGAGTCTTGTTGATAA	0.400 0.200 0.200	60°C 35 cycles	NAU and IPM^g^
Mad-62	1902978	G→A	s3-s9	s-s03	A^h^: CAGCATGCACCAGCCCaGG D: cgggttcgggttcggggttcggggttcggggCAGCATGCACCAGCCCtGA C^h^: GGATGATGCCGTCGGTATTTTC	0.800 0.200 0.200	60°C 31 cycles	NAU
Mad-64	2393962	T→C	s4-s9	s03-s04	A^h^: GACGCACGGTGTGACAATAAAgTT D: cggggttcggggttcggggttcggggGACGCACGGTGTGACAATAAAcTC C^h^: GCTTTTTGCATTTGGATTCTCCTT	0.800 0.200 0.200	64°C 35 cycles Note: double band on derived genotype	NAU
Mad-65	435838	C→T	s5-s9	s04-s05	A^h^: GCTGAGAAAATTATGACAACTTTATCgCC D: cgggttcgggttcgggttcgggCTGAGAAAATTATGACAACTTTATCcCT C^h^: TGCTGCACAGCATTTCACTACGG	0.300 0.200 0.200	60°C 30 cycles	NAU
Mad-74	1355526	G→T	s8/s9	s18-s08	A: GAACCATAATTCACAATAAAAgG D: cgggttcggggttcggggttcggggAAGAACCATAATTCACAATAAAAcT C: TATCAGTTCTAATTTGAATGTAAGG	0.800 0.200 0.200	51°C 33 cycles	NAU
Mad-78	1780902	C→T	s9	s08-s09	A: ATTGCCAAGCTATTTGTCcG D: cggggttcggggttcggggttcgggATAACATTGCCAAGCTATTTGTgTA C: GTGTCGTATGCGATAGCACTAATA	0.200 0.100 0.200	60°C 35 cycles	NAU

^a^SNP position and naming as published in Vogler et al. 2011, 2013, 2017.

^b^SNP states are presented according to their orientation in the Yp CO-92 reference genome; D: Derived SNP state; A: Ancestral SNP state.

^c^D: Derived; A: Ancestral; C: Common Reverse.

^d^Primer tails and antepenultimate/and or penultimate mismatch bases are in lower case.

^e^Ratios are skewed in some assays.

^f^Standard MgCl_2_ is 2 mM.

^g^MgCl_2_ reduced to 1.5 mM at IPM from the standard 2 mM used at NAU.

^h^Primer originally published in Vogler et al 2013.

### PCR conditions and size based genotype discrimination

Initial PCR conditions were identified at NAU. PCR conditions for different assays varied and are described in Table 3. PCR amplification per assay was carried out in 20 μL volume with the following reagents (see S1 Appendix for volume of each reagent): for one reaction, 1x PCR buffer without MgCl_2_, MgCl_2_ range of 1.5–2.5 mM, 0.30 mM deoxynucleoside triphosphate, 1.6 units of platinum *Taq* DNA polymerase (Invitrogen, Carlsbad, CA), both sets of forward MAMA primers (derived and ancestral allele-specific) with one common reverse primer at 0.40 μL each (for a 1:1 ratio), molecular grade water to achieve 18 μL total volume and 2.0 μL of diluted DNA template at ~1ng/μL per reaction. We did not directly test our assays on genomic DNA concentrations below 100 pg but previously published work suggests that the MAMA approach is sensitive to DNA amounts below 100 pg [20]. For each set of reactions, at least one of each ancestral and derived allele templates were used as positive controls as well as at least two no-template controls (NTC). Thermal cycling parameters for the eighteen assays are as follows: initial denaturation at 94°C for 5 min followed by 30–40 cycles of 94°C for 30 s, 51°C -67.3°C (Table 3) for 30 s, and 72°C for 30 s, with a final extension at 72°C for 5 min. All PCR amplifications were performed with a MJ Research PTC 200 thermal cycler (BioRad, Hercules, CA).

Conditions of agarose gel electrophoresis for PCR amplicons included adding 4 μL of 6x loading dye 0.25 w/v xylene cyanol FF and 30% v/v glycerol, water (Thermo Fisher scientific, Waltham, MA) to individual PCR products to achieve a 1x final dye concentration. Individual reactions (20 μL) mixed with loading dye were loaded onto a 2% agarose gel matrix; 100 bp DNA ladder (Invitrogen, Carlsbad, CA) was used for size referencing. Gels were prepared in 1x lithium borate buffer (S2 Appendix) and stained with SybrSafe dye (Life Technologies, Carlsbad, CA). Electrophoresis was conducted at 300V for 25–30 minutes and viewed under UV transillumination.

The genotyping accuracy of our SNP assays was validated using positive DNA controls that represented known ancestral and derived allele states for each SNP target. Assay accuracy was further assessed by testing them on 16 genomic DNA extracts of Malagasy *Y*. *pestis* strains belonging to known diverse phylogenetic groups (Table 1) based on amplicon sequencing [34]. To assess assay capability to genotype *Y*. *pestis* directly from complex DNA samples (containing high levels of host DNA), we tested the performance of each assay on a positive control sample comprised of high concentrations of human DNA with our positive control *Y*. *pestis* DNA extract (strain A1122). To further assess this capability, we tested two assays (Mad-05 and Mad-43) on five human complex samples confirmed to be plague positive. This confirmation was based on *Y*. *pestis*-specific TaqMan assay targeting a high copy plasmid *pla* gene [37]. We assessed the limit of detection of our MAMA tools on these five human clinical samples by testing them on a new TaqMan assay designed for the chromosomal *3a* gene (Fig 4 and S3 Appendix).

To confirm the specificity of the assays to the *Y*. *pestis* genome we tested assay performance on DNA extracts of *Bacillus anthracis*, human DNA background, and no template water control. To compare assay performance at different research facilities, NAU and IPM jointly conducted a second validation study on the IPM laboratory premises using IPM PCR reagents and instrument (Fig 5). Thirteen of the eighteen assays were selected for validation (Table 3). At IPM, specificity was confirmed by testing on *Leptospira interrogans* serovar *Canicola* and no template controls. PCR was prepared for each assay using the *Taq* Core Kits 10, Cat# EPTQK300 PCR reagents (MP Biomedicals, Santa Ana, CA) and amplification was conducted using AB Applied Biosystems Veriti 96 Well Thermal Cycler thermal cycle (ThermoFisher Scientific, Waltham, MA). Electrophoresis was performed using 2% agarose gels visualized on a Gelscan (Bio-Rad, Hercules, CA).

## Supporting information

S1 Appendix PCR master mix calculation sheet indicating final concentration and volume addition of each reagent. (DOCX)

S2 Appendix Side by side comparison of 2% agarose gel electrophoresis in a 1x Lithium Borate and 1x TAE matrix. Electrophoresis was conducted with identical PCR products, equipment and agarose gel conditions. The two runs differ only in buffer solution used.
(DOCX)

S3 Appendix *Y*. *pestis* 3a TaqMan assay design. (DOCX)

S4 Appendix SNP allele state for the 16 isolates shown in Table 1. (DOCX)

S5 Appendix Agarose gel pictures for each assays generated at both NAU and IPM or NAU only. (DOCX)

S6 Appendix Agarose gel pictures of negative controls (human background DNA and *Bacillus anthracis*–A0635 and A0643) on a subset of MAMA tools. (DOCX)
