# Supplementary material for: Low cost, low tech SNP genotyping tools for resource-limited areas: Plague in Madagascar as a model
Source: PLoS Negl Trop Dis. 2017 Dec 11;11(12):e0006077. doi: 10.1371/journal.pntd.0006077 (PMC5739503; doi:10.1371/journal.pntd.0006077)

**Supporting Information**

**S1 Appendix. PCR master mix calculation sheet indicating final concentration and volume addition of each reagent.**


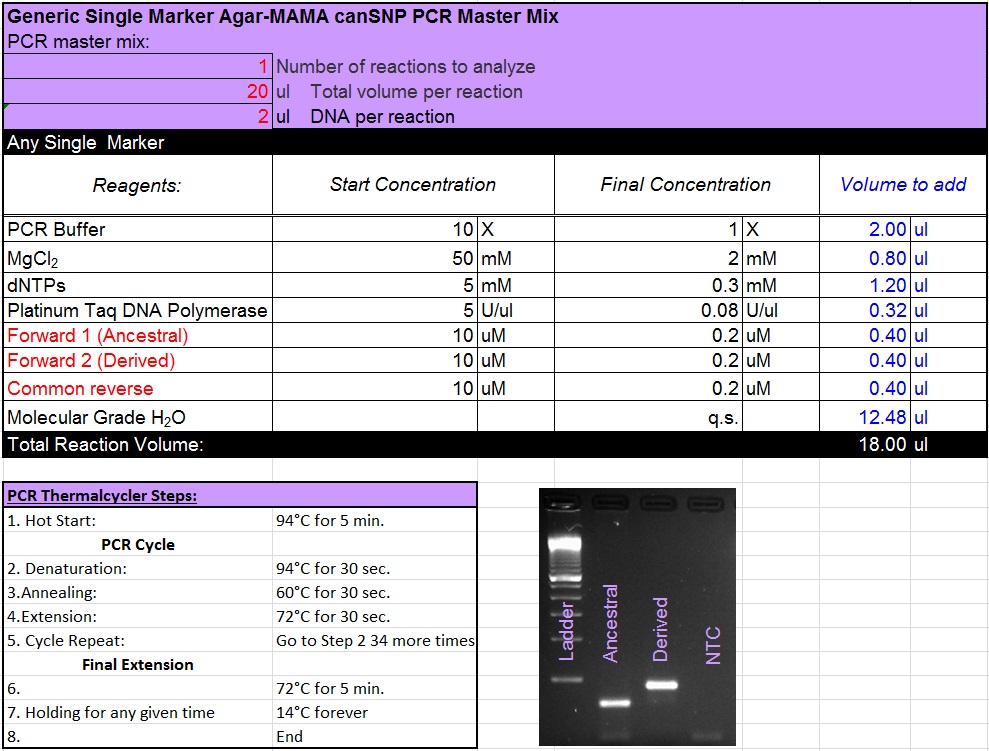

Supplement: S1 Appendix — (DOCX) [file pntd.0006077.s001.docx]
