# Supplementary material for: Low cost, low tech SNP genotyping tools for resource-limited areas: Plague in Madagascar as a model
Source: PLoS Negl Trop Dis. 2017 Dec 11;11(12):e0006077. doi: 10.1371/journal.pntd.0006077 (PMC5739503; doi:10.1371/journal.pntd.0006077)
Supplement: S2 Appendix — Electrophoresis was conducted with identical PCR products, equipment and agarose gel conditions. The two runs differ only in buffer solution used. (DOCX) [file pntd.0006077.s002.docx]

**Supporting Information**

**S2 Appendix. Side by side comparison of 2% agarose gel electrophoresis in a 1x Lithium Borate and 1x TAE matrix.** Electrophoresis was conducted with identical PCR products, equipment and agarose gel conditions. The two runs differ only in buffer solution used.


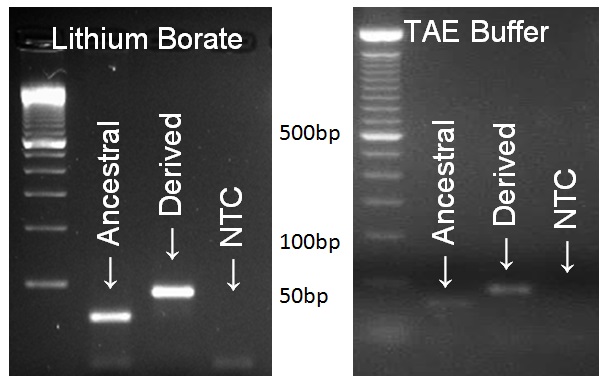


**Side by side comparison of 2% agarose gel electrophoresis in a 1x Lithium Borate and 1x TAE matrix**

Lithium borate buffer was prepared using lithium hydroxide monohydrate 56% cat. # AA1340936 (Fisher Scientific, Waltham, MA), boric acid and distilled water in 1L batches at a 20x concentration. In a fume hood, 850mL of distilled water was initially added to a 1L glass beaker. Water was kept at a low heat and stirred at a medium speed. Slowly, 8.2g of lithium hydroxide monohydrate was added to the beaker followed by ~ 40g of boric acid. The solution was heated and stirred until fully dissolved, then allowed to cool. At room temperature the pH was measured and boric acid was added until the pH reached 8.2. Distilled water was added to reach a 1L volume and the solution was filter-sterilized and stored in a 1L plastic container as lithium hydroxide can be corrosive to glass over time. For electrophoresis use, the 20x lithium borate stock was diluted to a 1x final concentration using distilled water.
