# Supplementary material for: Low cost, low tech SNP genotyping tools for resource-limited areas: Plague in Madagascar as a model
Source: PLoS Negl Trop Dis. 2017 Dec 11;11(12):e0006077. doi: 10.1371/journal.pntd.0006077 (PMC5739503; doi:10.1371/journal.pntd.0006077)
Supplement: S3 Appendix — (DOCX) [file pntd.0006077.s003.docx]

**Supporting Information**

**S3 Appendix. *Y. pestis* 3a TaqMan assay design.**

The *Y. pestis*-specific target (3a) is found in single copy on the *Y. pestis* chromosome. The primers and probe were designed based on the primers and sequence of 3a. [[1](#_ENREF_1)]. Yp3aF-CATTGGACGGCATCACGAT, Yp3aR- AGTTGGCCAGCGATTCGA, Yp3aPROBE- VIC-CTCTACCAAAACGCC. Real-time PCR assays contained 1x AB TaqMan® Universal PCR Master Mix with AmpErase UNG (Life Technologies, Foster City, CA), Yp3a F and Yp3aR primers (final concentration 0.60 µM), Yp*3a* FAM-labeled probe (final concentration 0.25µM), and 1µL of DNA template. The samples were run in a final reaction volume of 10µL under the following conditions: 95°C for 10 minutes, 40 cycles of 95°C for 30 seconds and 60°C for 1 minute. The resulting fluorescent amplicons were detected using ABI 7900HT Fast Real-time PCR System (Life Technologies, Foster City, CA).

1. Radnedge L, Gamez-Chin S, McCready PM, Worsham PL, Andersen GL (2001) Identification of nucleotide sequences for the specific and rapid detection of Yersinia pestis. Appl Environ Microbiol 67: 3759-3762.
