# Supplementary material for: Low cost, low tech SNP genotyping tools for resource-limited areas: Plague in Madagascar as a model
Source: PLoS Negl Trop Dis. 2017 Dec 11;11(12):e0006077. doi: 10.1371/journal.pntd.0006077 (PMC5739503; doi:10.1371/journal.pntd.0006077)
Supplement: S4 Appendix — (DOCX) [file pntd.0006077.s004.docx]

**Supporting Information**

**S4 Appendix. Table showing the SNP allele state for the 16 isolates shown in Table 1.**

| **ID^a^** | **Original ID^b^** | **Group^c^** | **Vogler et al. 2013 SNP Node*^d^*** | ***Mad-05 (s53) C→T** | **Mad-12 (s1376) G→A** | **Mad-08 (s1362) C→T** | **Mad-58 A→G** | **^e^Mad-62 G→A** | **^e^Mad-64 T→C** | **^e^Mad-65 C→T** | **^e^Mad-74 G→T** | **^e^Mad-78 C→T** | **Mad-30 G→A** | **Mad-32 (s298) T→A** | **Mad-35 (s910) G→A** | **Mad-36 (s374) C→T** | **Mad-37 (s1389) G→A** | **Mad-38 (s641) A→G** | **Mad-42 (s1377) T→C** | **Mad-43 (s1089) C→T** | **Mad-46 (s1373) T→C** |
| --- | --- | --- | --- | --- | --- | --- | --- | --- | --- | --- | --- | --- | --- | --- | --- | --- | --- | --- | --- | --- | --- |
| Yp2568 | 119/96 | II | h1 | T | G | C | A | G | T | C | G | C | G | T | G | C | G | A | T | T | T |
| Yp2572 | 154/96 | II | h1 | T | G | C | A | G | T | C | G | C | G | T | G | C | G | A | T | T | T |
| Yp3070 | 87/11 | I | j | T | G | T | A | G | T | C | G | C | G | T | G | C | G | A | T | C | T |
| Yp2672 | 32/99 | I | l1 | T | A | C | A | G | T | C | G | C | G | T | G | C | G | A | T | C | T |
| Yp2554 | 194/95 | I | q5 | T | G | C | A | G | T | C | G | C | A | A | A | T | A | A | T | C | T |
| Yp2610 | 402/97 | I | q6 | T | G | C | A | G | T | C | G | C | A | A | A | T | G | G | T | C | T |
| Yp2924 | 26/05 | I | q6 | T | G | C | A | G | T | C | G | C | A | A | A | T | G | G | T | C | T |
| Yp2975 | 145/06 | I | q2 | T | G | C | A | G | T | C | G | C | A | A | G | C | G | A | T | C | T |
| Yp2564 | 67/96 | I | r | T | G | C | A | G | T | C | G | C | G | T | G | C | G | A | C | C | T |
| Yp2675 | 63/99 | I | s3 | T | G | C | G | A | T | C | G | C | G | T | G | C | G | A | T | C | T |
| Yp2933 | 71/05 | I | s5 | T | G | C | G | A | C | *^f^*T | G | C | G | T | G | C | G | A | T | C | T |
| Yp3020 | 02/08 | I | s5 | T | G | C | G | A | C | *^f^*T | G | C | G | T | G | C | G | A | T | C | T |
| Yp2669 | 172/98 | I | s9 | T | G | C | G | A | C | T | T | T | G | T | G | C | G | A | T | C | T |
| Yp2559 | 17/96 | II | d | T | G | C | A | G | T | C | G | C | G | T | G | C | G | A | T | T | C |
| Yp2560 | 19/96 | II | d | T | G | C | A | G | T | C | G | C | G | T | G | C | G | A | T | T | C |
| Yp2561 | 26/96 | II | d | T | G | C | A | G | T | C | G | C | G | T | G | C | G | A | T | T | C |

*^a^*Strain ID in the Northern Arizona University DNA collection.

*^b^*Strain ID from the originating laboratory.

*^c^*Indicates Group I or II, as determined by SNP separating nodes d and k in Fig 1 (Vogler et al 2017)

*^d^*Indicates SNP determined node (Vogler et al 2011 and Vogler et al 2013).

*^e^*Agarose-MAMA tools not tested at IPM (as indicated on Table 3). SNP state for each isolate confirmed with Agarose-MAMA at NAU and by sequencing at Vogler et al 2017.

*^f^*SNP state for isolate confirmed by Agarose-MAMA at NAU and by sequencing in Vogler et al 2017 but not tested at IPM.
