# Supplementary material for: Low cost, low tech SNP genotyping tools for resource-limited areas: Plague in Madagascar as a model
Source: PLoS Negl Trop Dis. 2017 Dec 11;11(12):e0006077. doi: 10.1371/journal.pntd.0006077 (PMC5739503; doi:10.1371/journal.pntd.0006077)
Supplement: S5 Appendix — (DOCX) [file pntd.0006077.s005.docx]

**Supporting Information**

**S5 Appendix. Agarose gel pictures for each assays generated at both NAU and IPM or NAU only.**

**
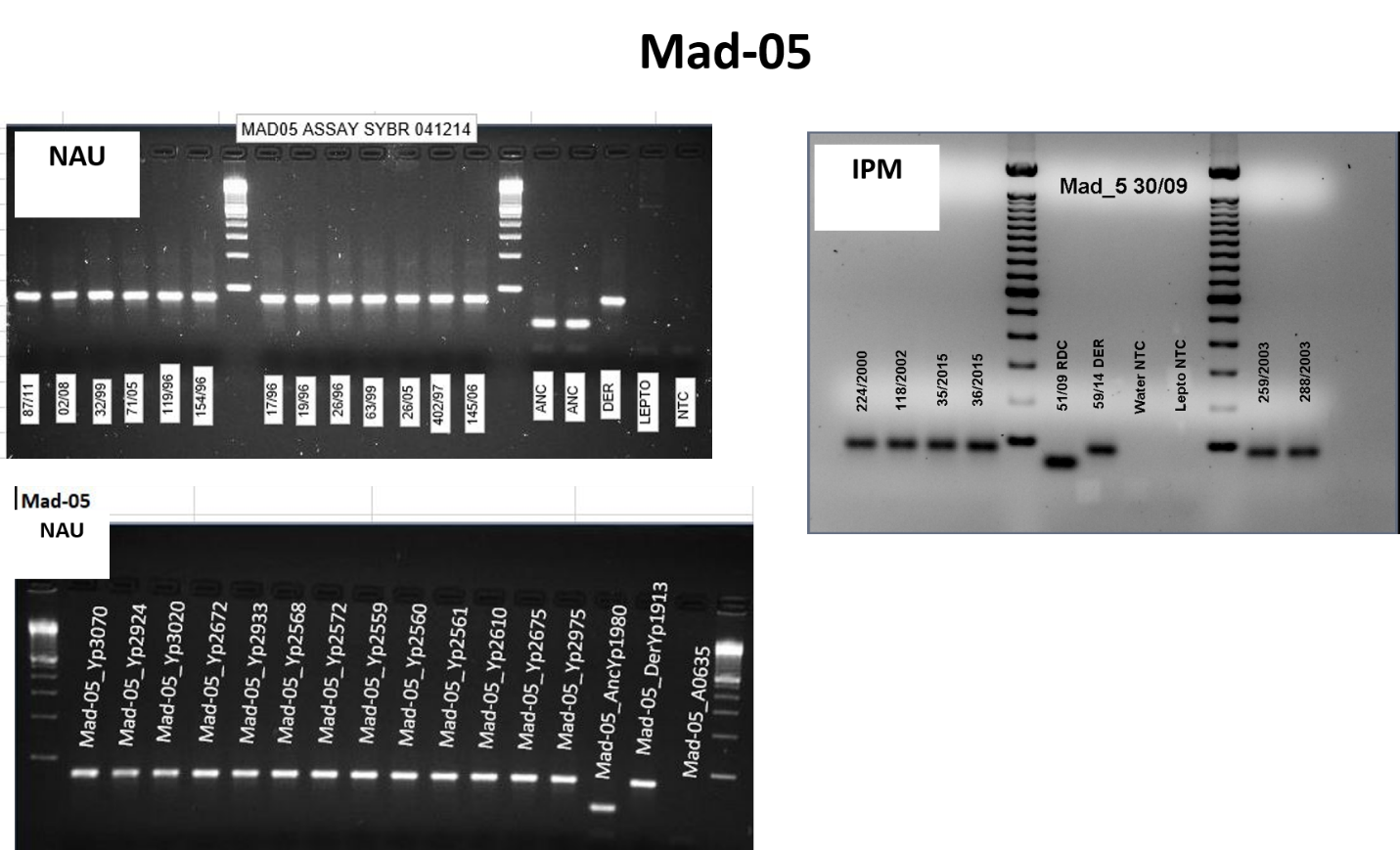
**

**
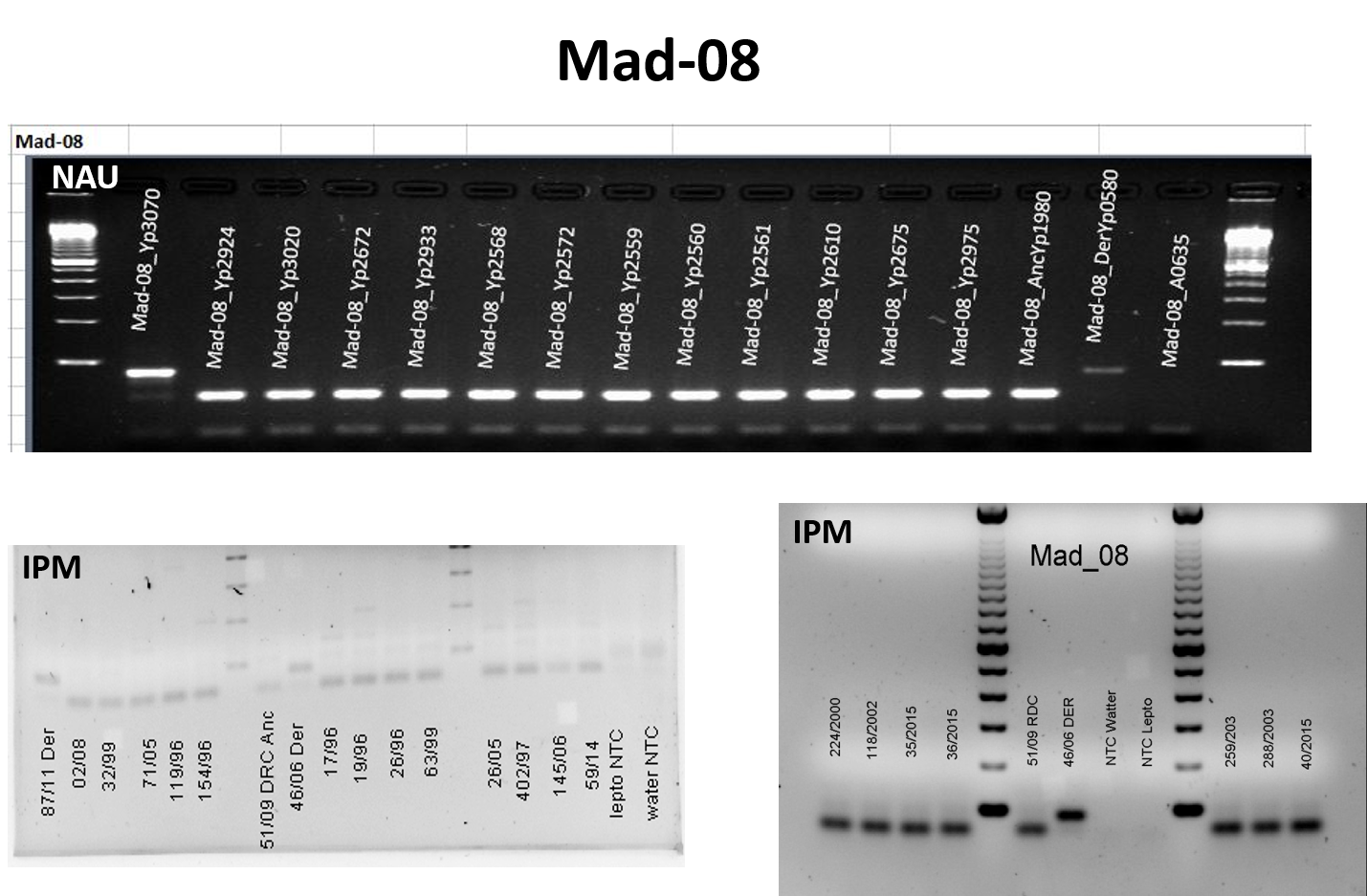
**

**
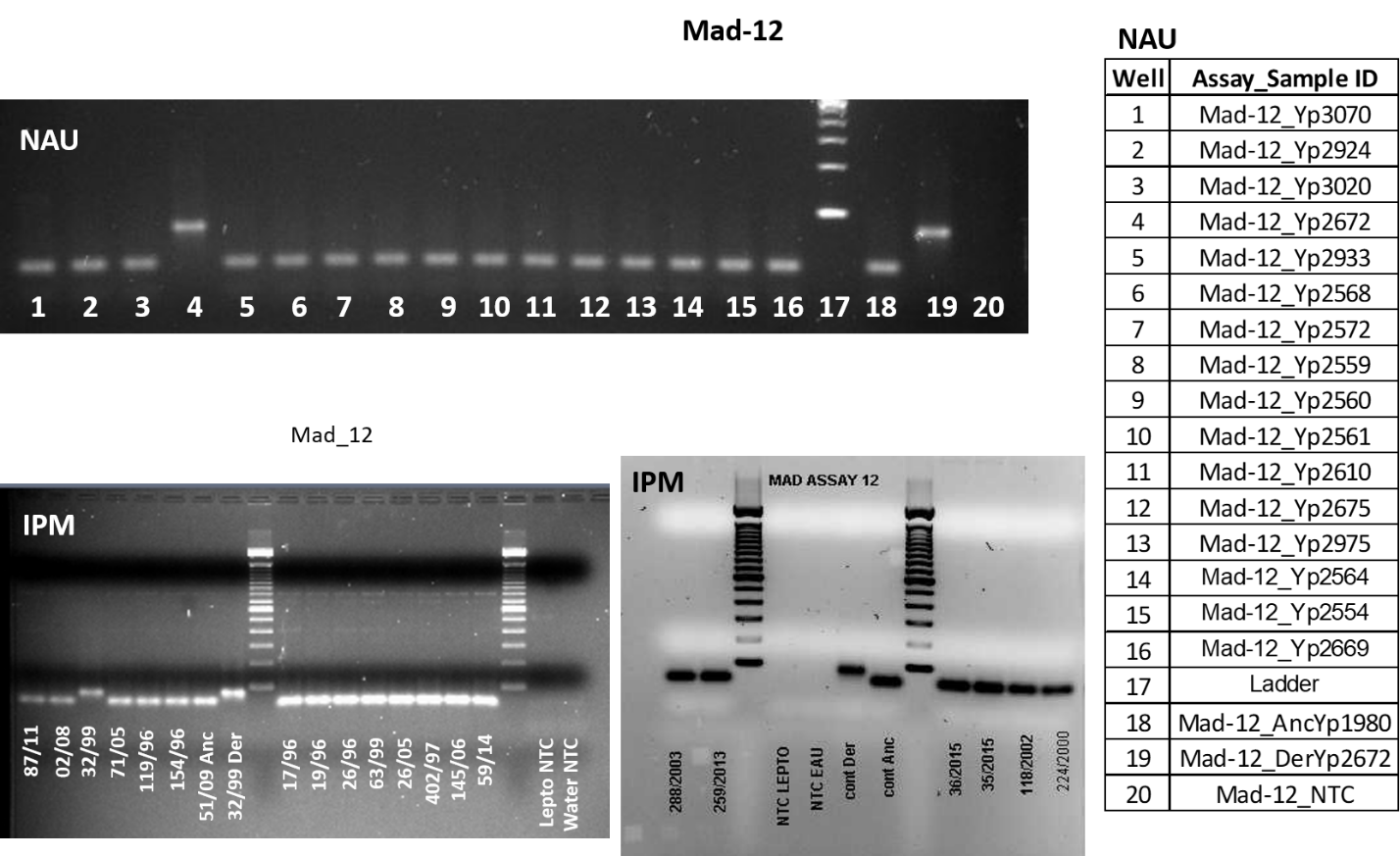
**

**
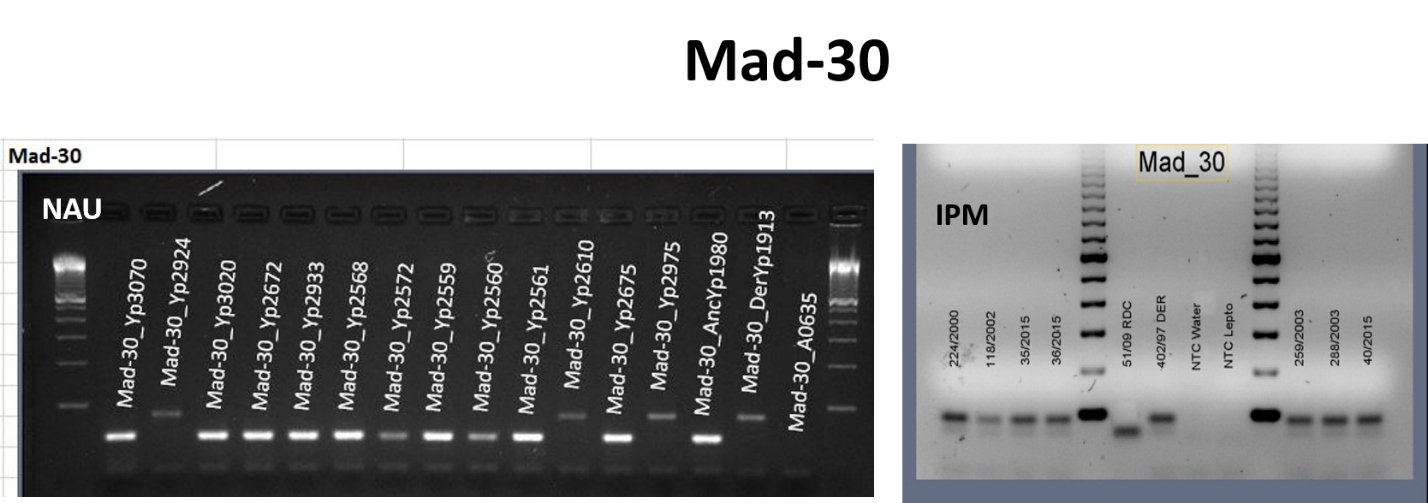
**

**
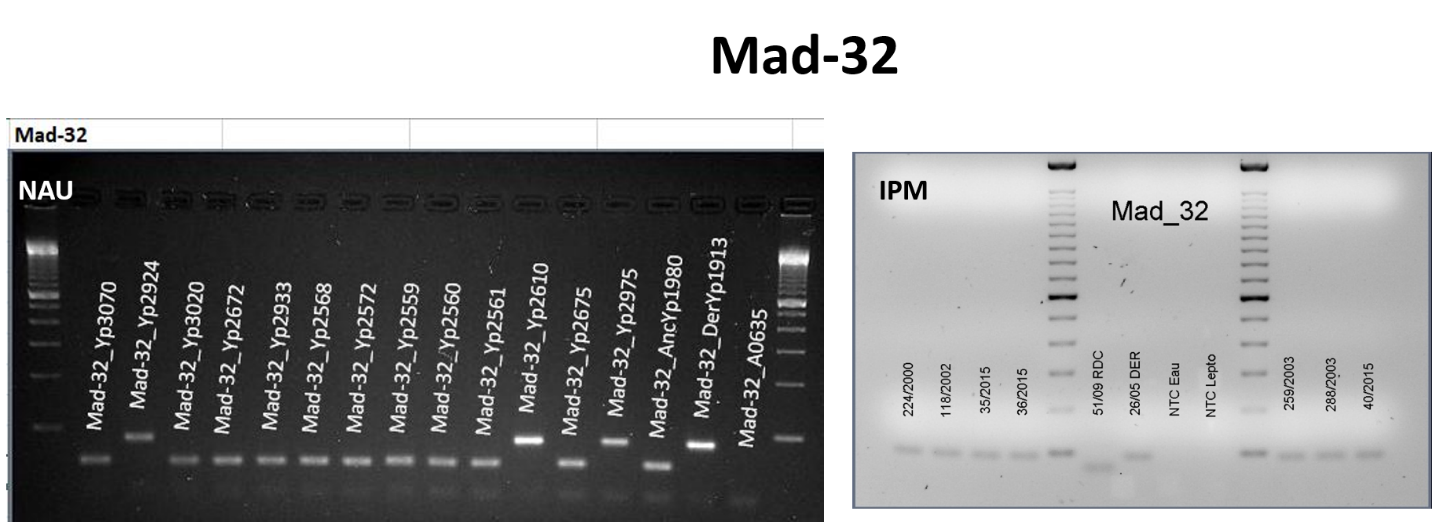
**

**
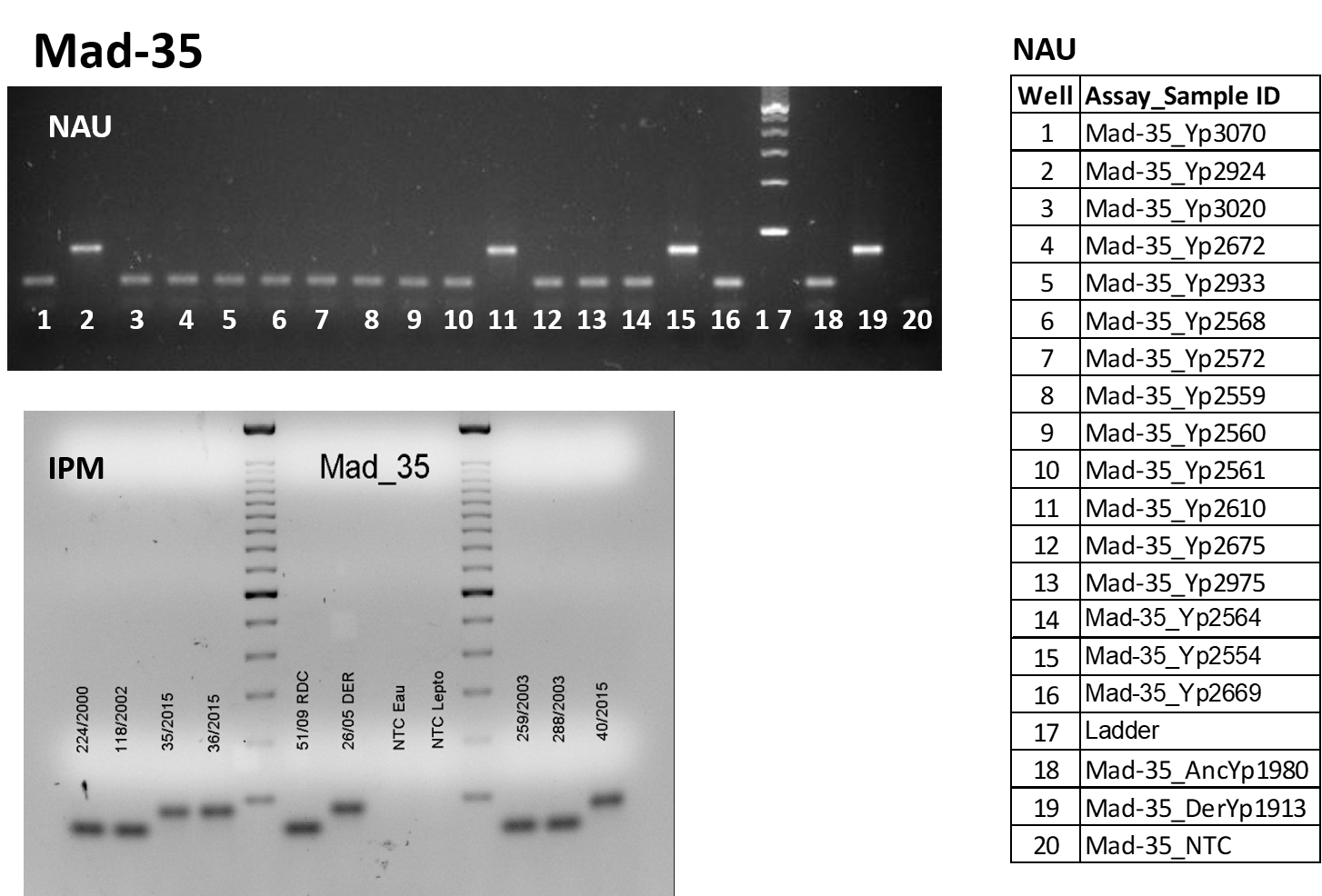
**

**
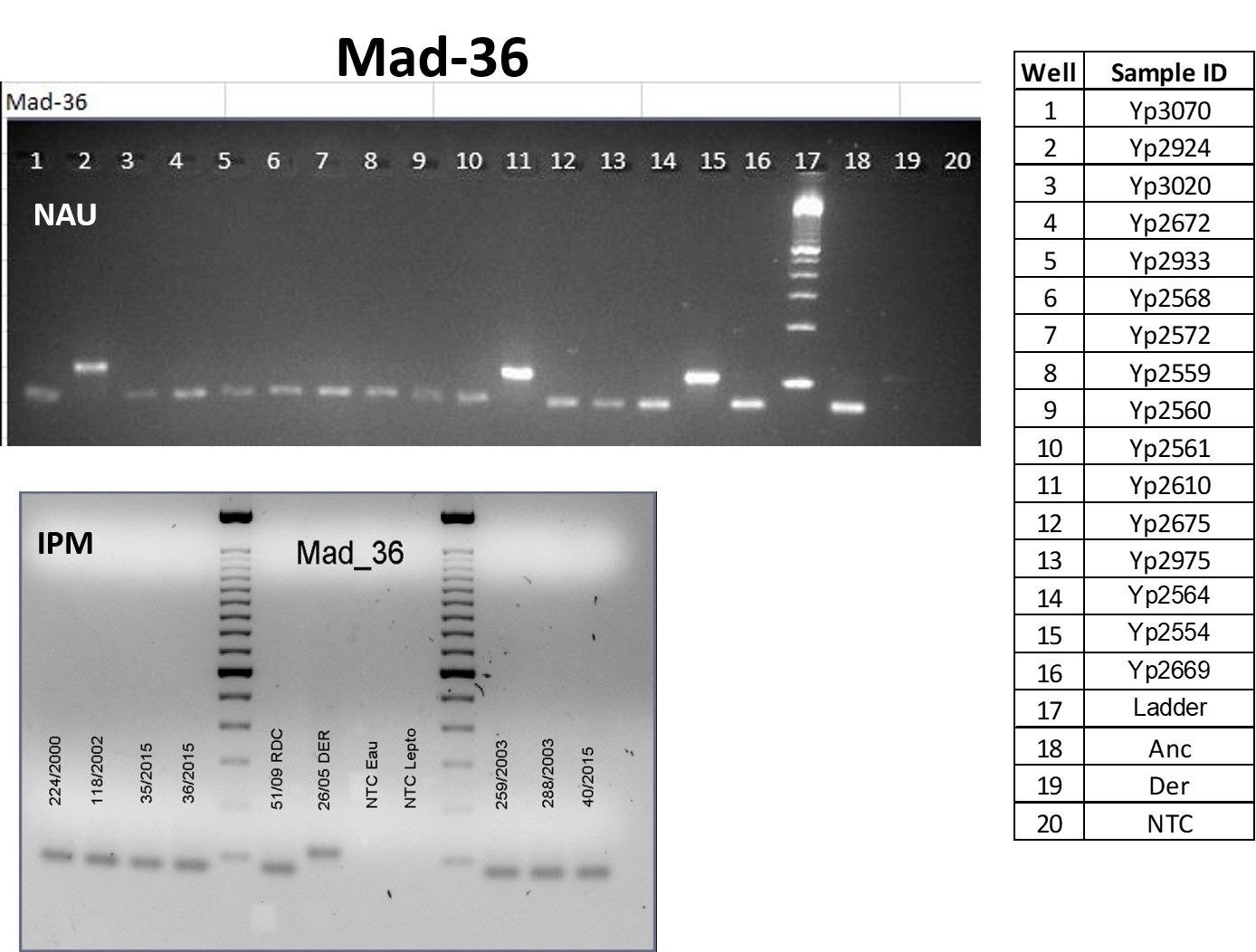
**

**
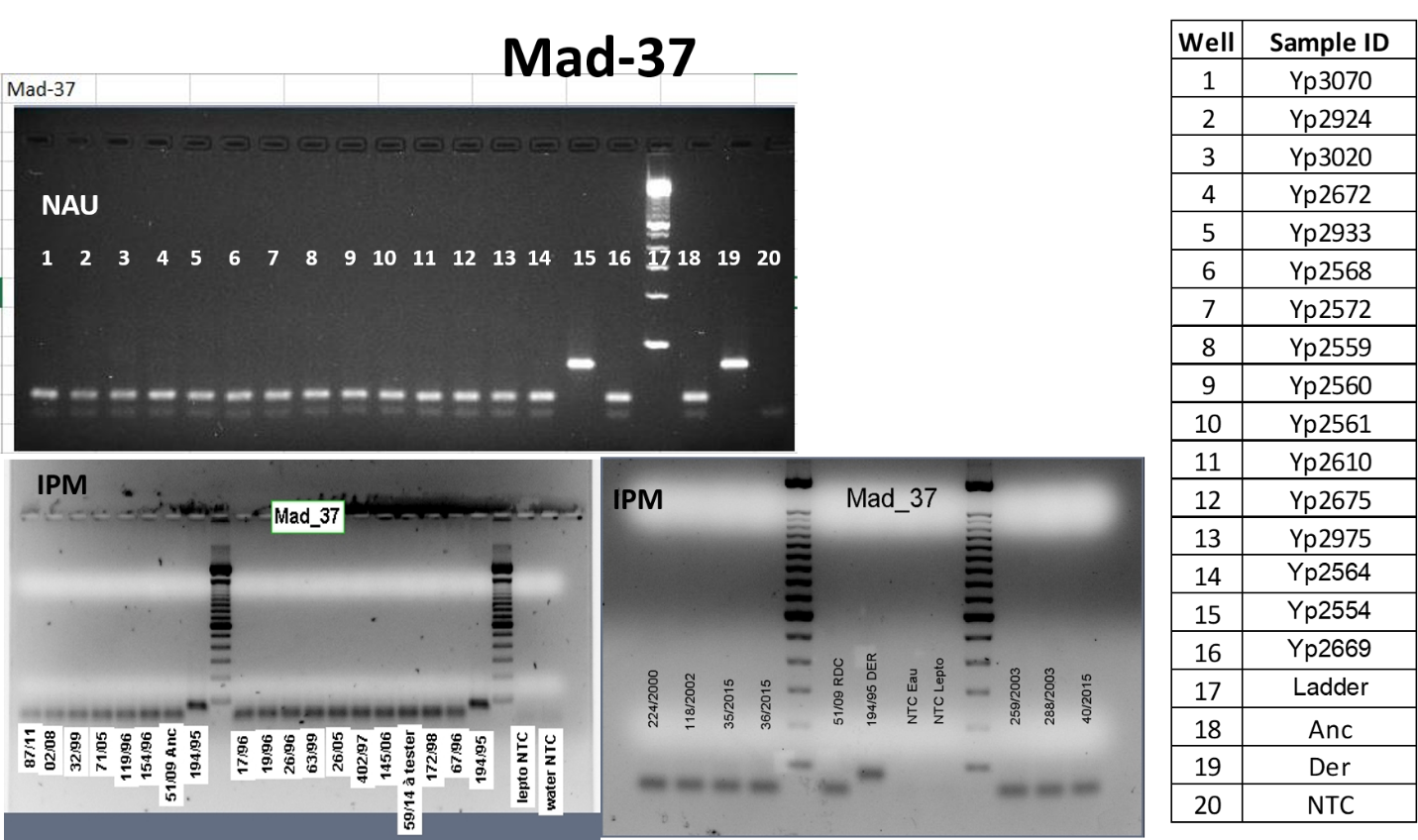
**

**
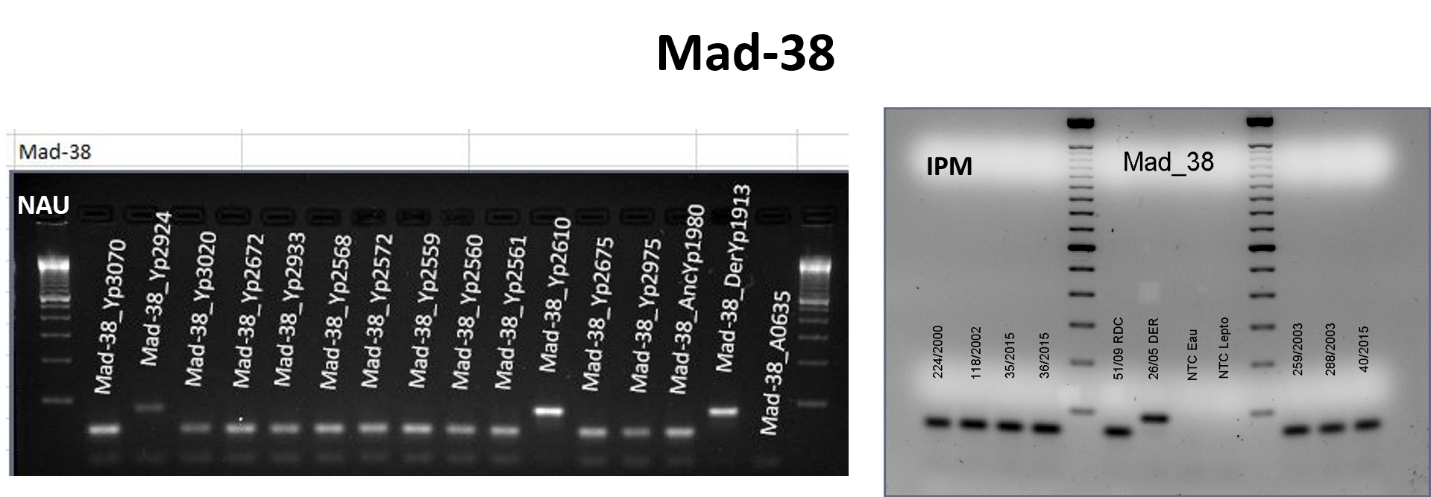
**

**
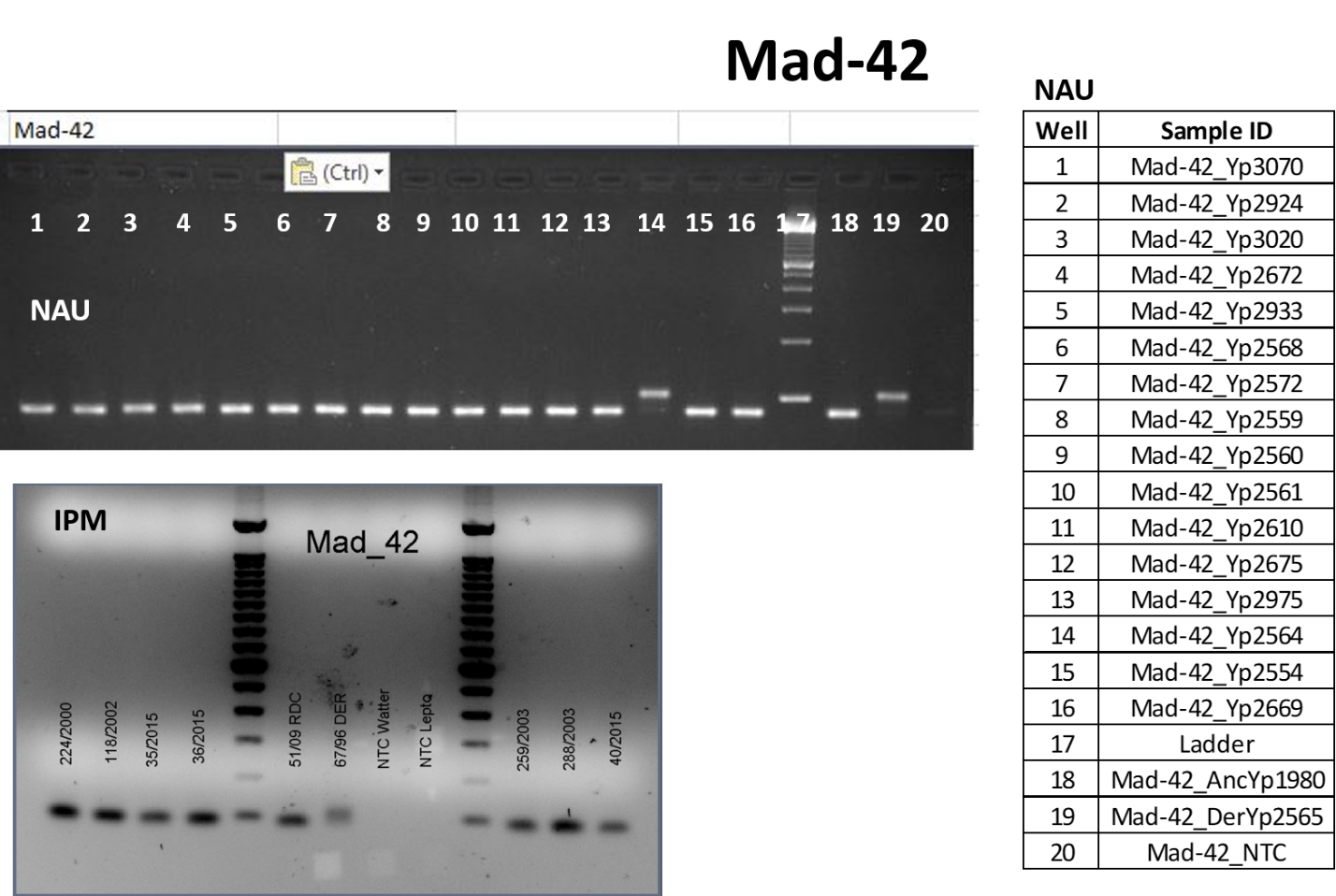
**

**
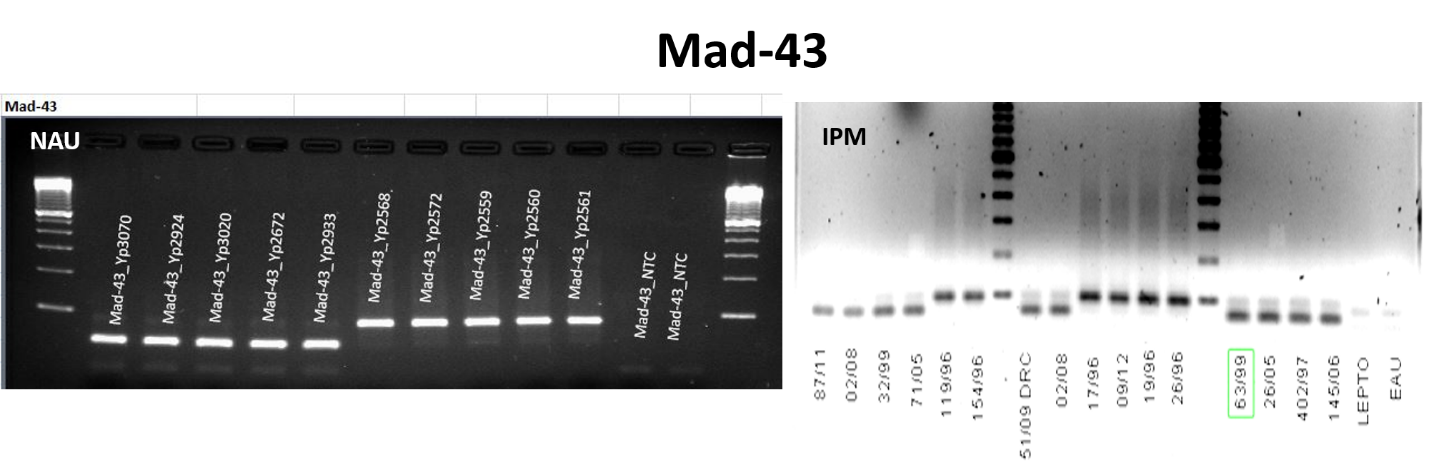
**

**
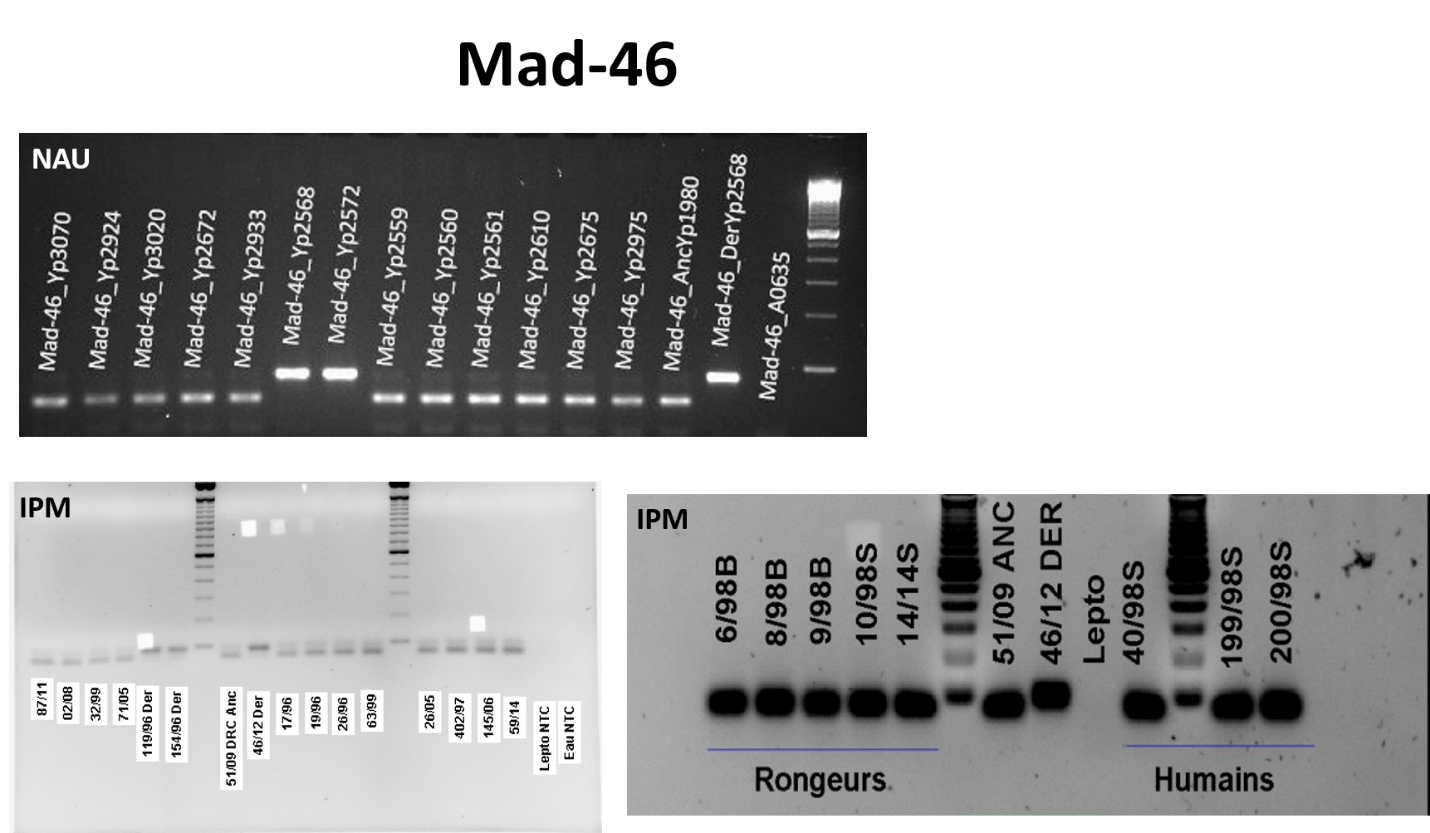
**

**
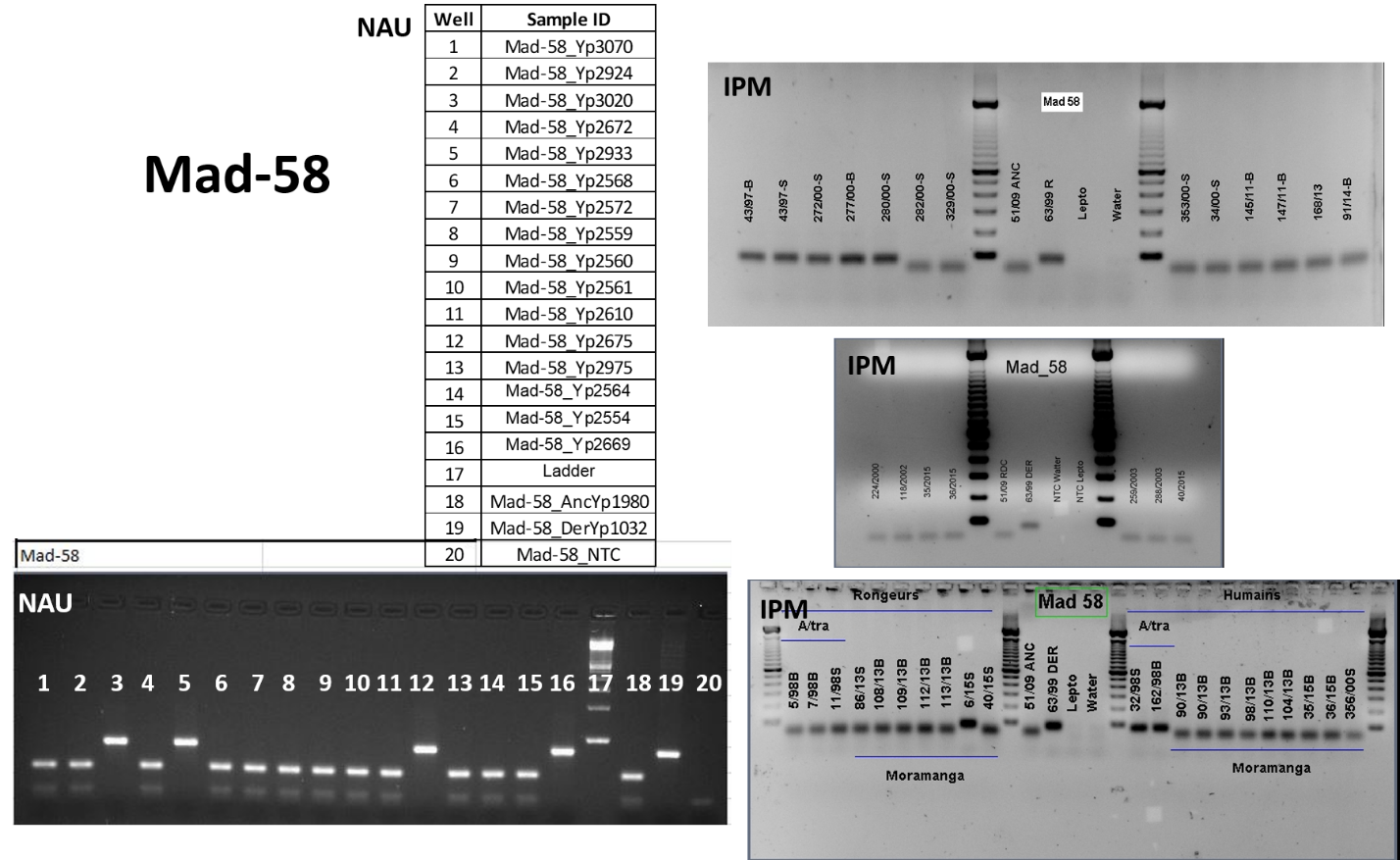
**

**Assays validated at NAU only**

**
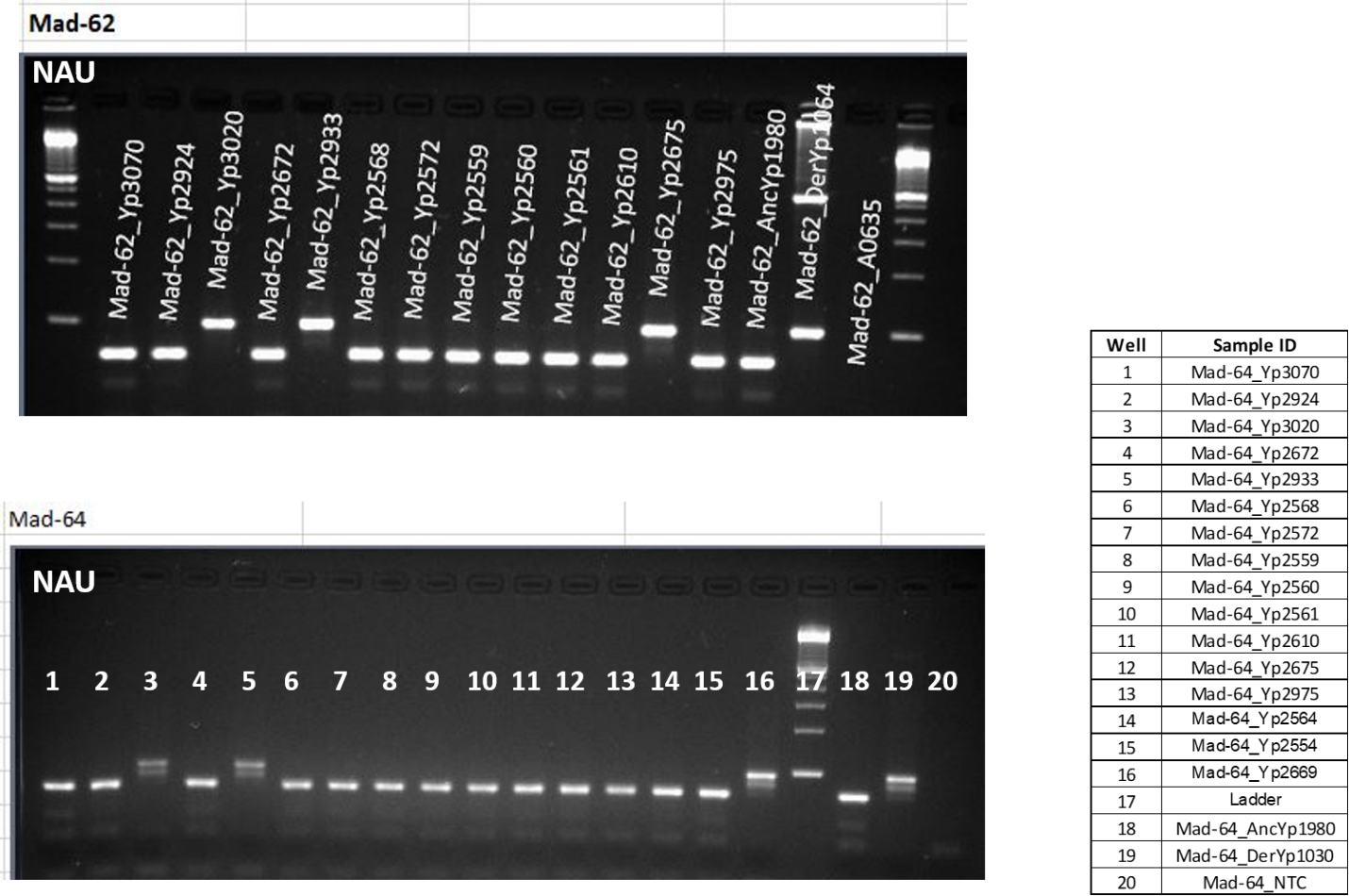
**

**
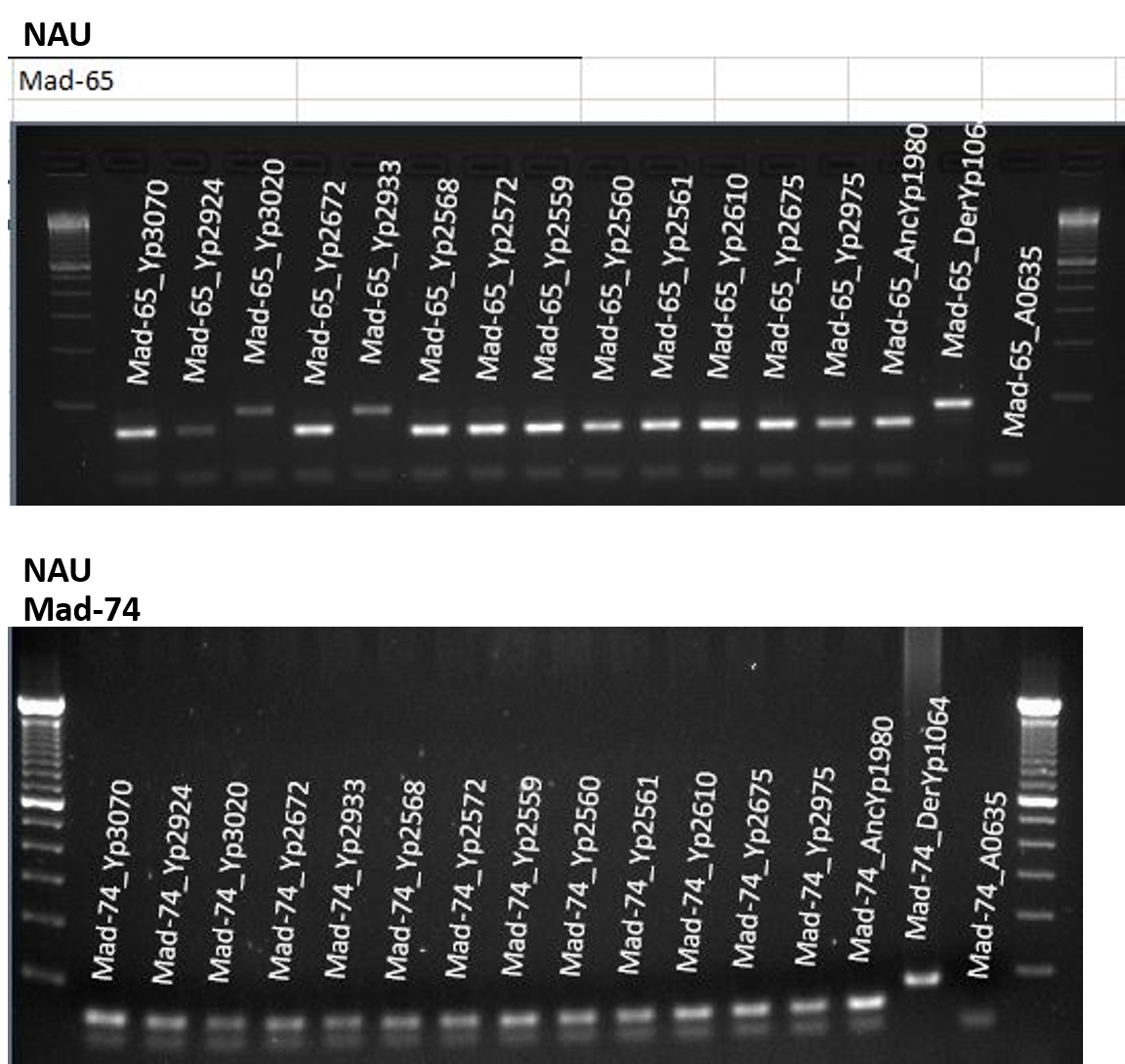
**

**
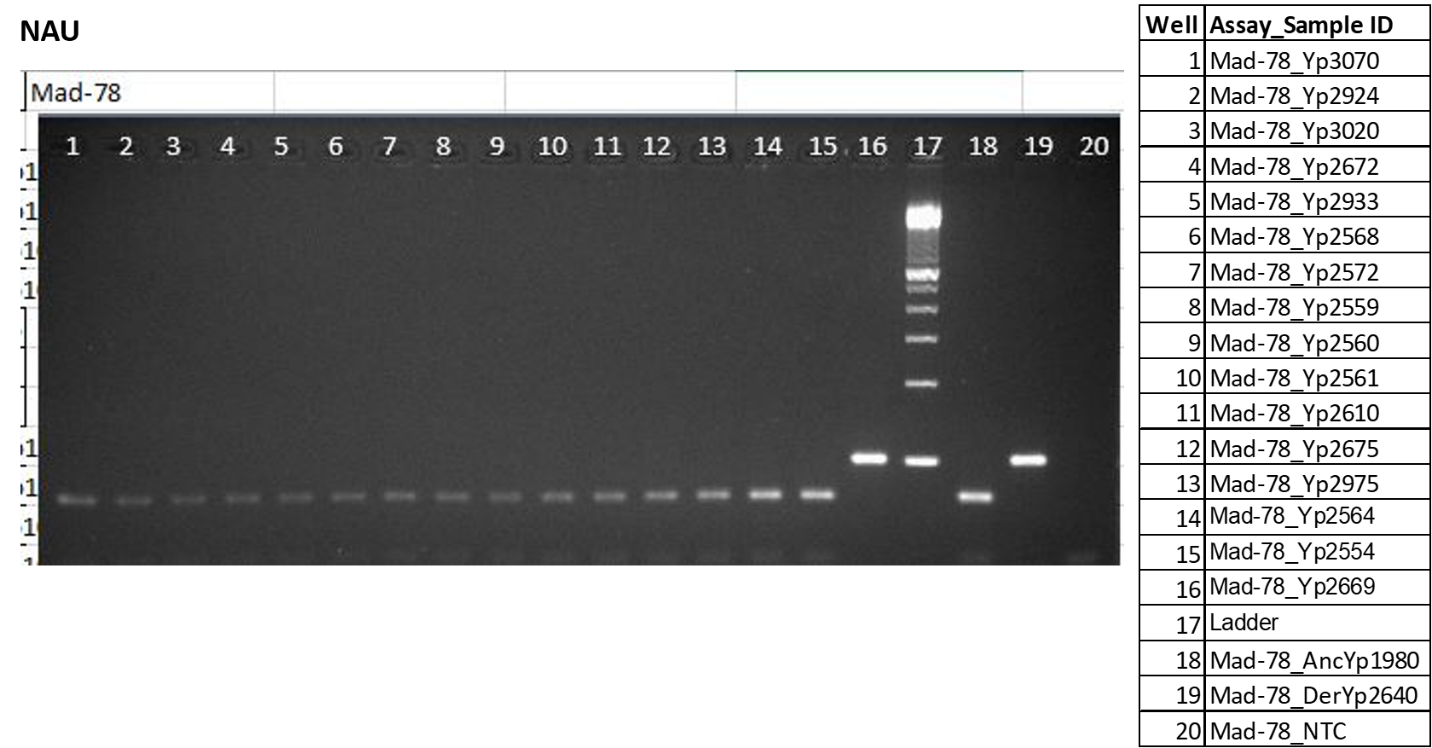
**

**Reference**

1. Vogler A, Andrianaivoarimanana V, Telfer S, Hall C, Sahl J, et al. (2017) Phylotemporalgeography of Yersinia pestis in Madagascar: Insight into the Long-Term Maintenance of Plague. PLoS Negl Trop Dis.

2. Radnedge L, Gamez-Chin S, McCready PM, Worsham PL, Andersen GL (2001) Identification of nucleotide sequences for the specific and rapid detection of Yersinia pestis. Appl Environ Microbiol 67: 3759-3762.
