# Supplementary material for: Low cost, low tech SNP genotyping tools for resource-limited areas: Plague in Madagascar as a model
Source: PLoS Negl Trop Dis. 2017 Dec 11;11(12):e0006077. doi: 10.1371/journal.pntd.0006077 (PMC5739503; doi:10.1371/journal.pntd.0006077)

**Supporting Information**

**S6 Appendix. Agarose gel pictures of negative controls (human background DNA and *Bacillus anthracis* – A0635 and A0643) on a subset of MAMA tools.**


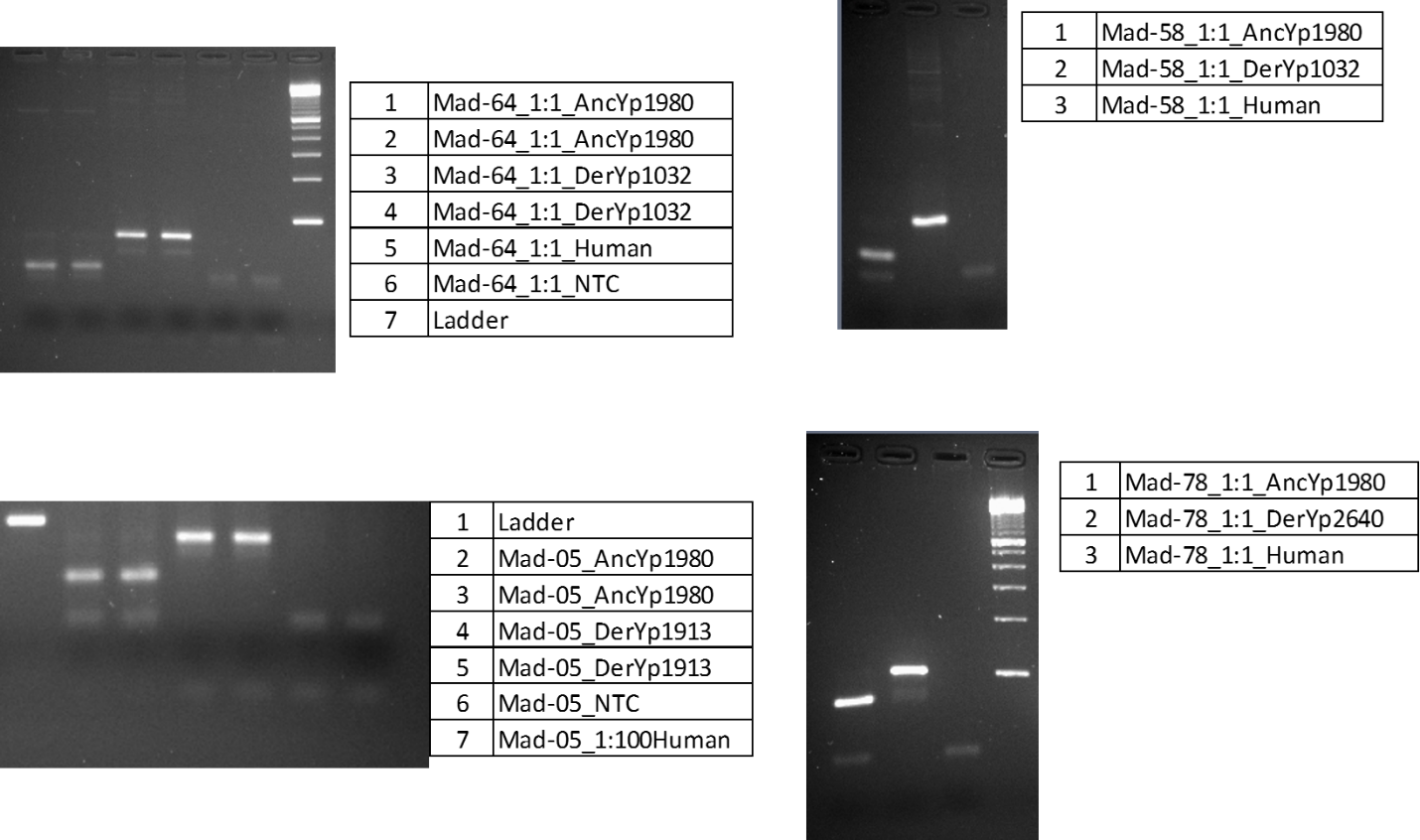


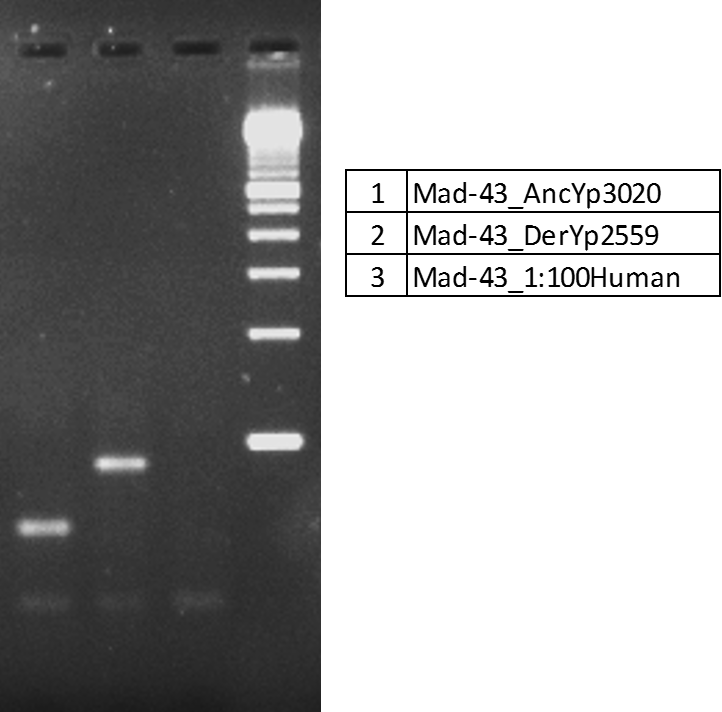


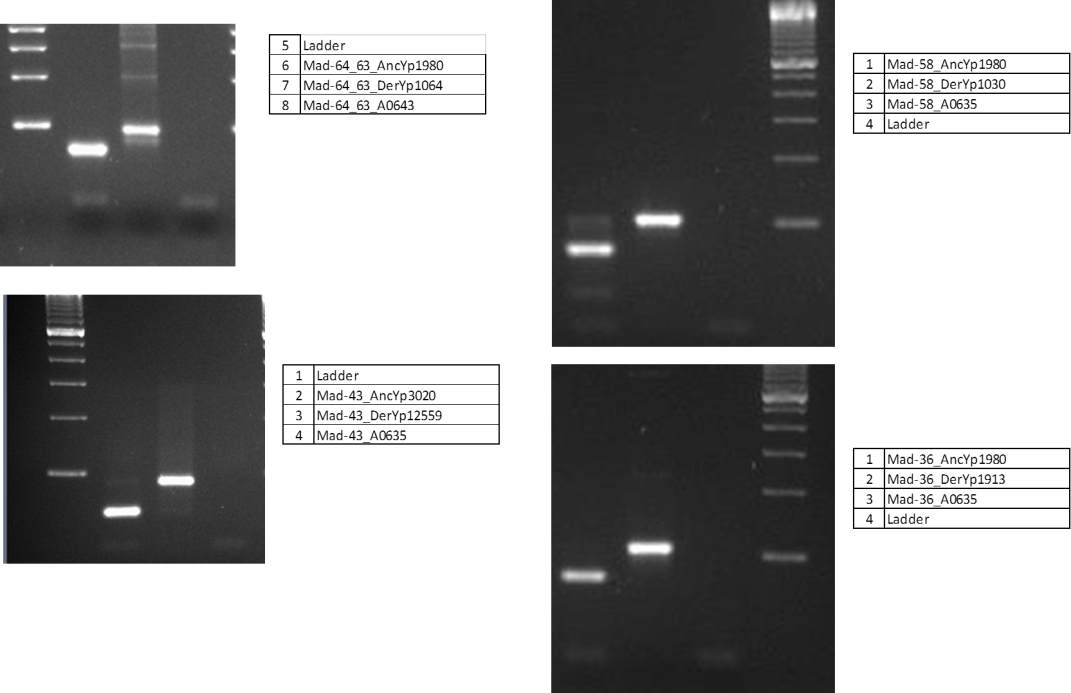


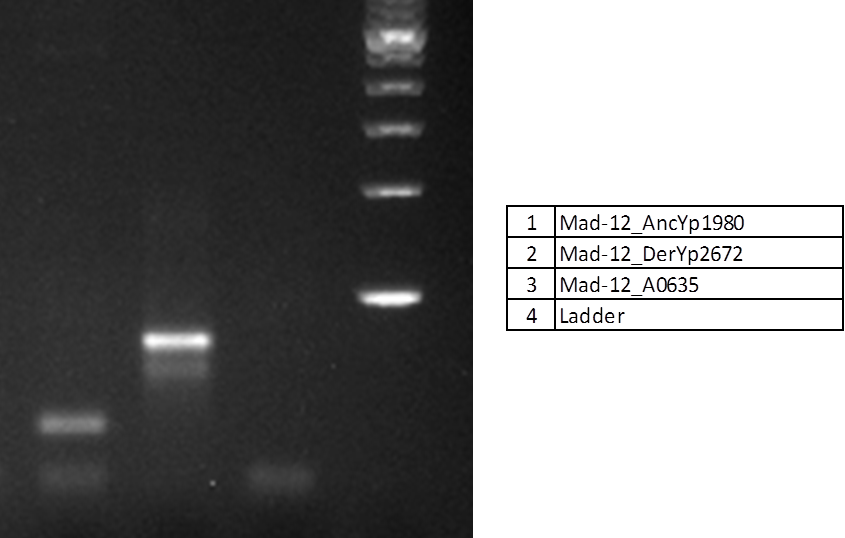

Supplement: S6 Appendix — (DOCX) [file pntd.0006077.s006.docx]
